# Major surgery induces acute changes in measured DNA methylation associated with immune response pathways

**DOI:** 10.1038/s41598-020-62262-x

**Published:** 2020-04-01

**Authors:** Ryoichi Sadahiro, Bridget Knight, Ffion James, Eilis Hannon, John Charity, Ian R. Daniels, Joe Burrage, Olivia Knox, Bethany Crawford, Neil J. Smart, Jonathan Mill

**Affiliations:** 1 Department of Immune Medicine, National Cancer Center Research Institute, National Cancer Center Japan, Tokyo, Japan; 2 University of Exeter Medical School, University of Exeter, Exeter, United Kingdom; 3 Royal Devon & Exeter Hospital, Exeter, United Kingdom

**Keywords:** DNA methylation, Biomarkers, Biomarkers

## Abstract

Surgery is an invasive procedure evoking acute inflammatory and immune responses that can influence risk for postoperative complications including cognitive dysfunction and delirium. Although the specific mechanisms driving these responses have not been well-characterized, they are hypothesized to involve the epigenetic regulation of gene expression. We quantified genome-wide levels of DNA methylation in peripheral blood mononuclear cells (PBMCs) longitudinally collected from a cohort of elderly patients undergoing major surgery, comparing samples collected at baseline to those collected immediately post-operatively and at discharge from hospital. We identified acute changes in measured DNA methylation at sites annotated to immune system genes, paralleling changes in serum-levels of markers including C-reactive protein (CRP) and Interleukin 6 (IL-6) measured in the same individuals. Many of the observed changes in measured DNA methylation were consistent across different types of major surgery, although there was notable heterogeneity between surgery types at certain loci. The acute changes in measured DNA methylation induced by surgery are relatively stable in the post-operative period, generally persisting until discharge from hospital. Our results highlight the dramatic alterations in gene regulation induced by invasive surgery, primarily reflecting upregulation of the immune system in response to trauma, wound healing and anaesthesia.

## Introduction

Surgery is an invasive procedure that elicits dramatic challenges to normal regulatory and homeostatic processes in the body. Importantly, surgery evokes acute inflammatory and immunological responses, which result directly from the trauma of surgery itself that acts to modulate wound healing and other physiological pathways^1–3^. Of note, changes to the immunological milieu following major surgery are thought to mediate risk for a number of postoperative complications including nosocomial infections^4^, and postoperative cognitive dysfunction and delirium^5^.

Post-operatively the immune system acts to promote tissue regeneration by influencing signal transduction, cell migration, differentiation, proliferation, and tissue remodelling^6,7^. Although the specific mechanisms driving these responses have not been well-characterized, they are hypothesized to involve the epigenetic regulation of gene expression^8^. Epigenetic processes act to dynamically control transcription via modifications to DNA, histone proteins, and chromatin acting independently of DNA sequence variation, and are known to regulate the function of immune cells during health and disease^9^. Of note, changes in DNA methylation have been reported to mediate postsurgical pain sensitivity^10^ and response to anaesthesia^11,12^ and opioids^13,14^.

In this study we explored the hypothesis that immunological modulation following surgery is associated with acute changes in DNA methylation. We longitudinally profiled DNA methylation in PBMCs collected from a cohort of elderly patients undergoing different types of major surgery at three time-points. Our study identifies dramatic shifts in DNA methylation in the vicinity of genes involved in immune regulation, with these changes being relatively stable across the post-operative period.

## Results

### Methodological overview

Using a longitudinal study design, we quantified DNA methylation across the genome in a cohort of 30 elderly patients (average age = 77.9 years (range 62–91)) undergoing major surgery (either elective colorectal surgery, elective hip replacement surgery, and emergency hip surgery following fracture). From each individual, detailed clinical blood measures were collected and DNA methylation was profiled using the Illumina Human Methylation 450 array (“450 K array”) (Illumina Inc. San Diego, California) in DNA samples isolated from PBMCs collected at three time-points: (i) immediately before surgery (baseline (BL)), (ii) in the morning of post-operative day 1 (POD1) and (iii) between post-operative day 4 and 7 (POD4/7) prior to discharge from hospital. Our analyses focused on identifying differentially methylated positions (DMPs) and regions (DMRs) reflecting acute changes induced by major surgery (see Methods). An overview of the study design and sampling strategy is given in Supplementary Fig. S1 and details about the individual patients included in this study are presented in Table 1.

**Table 1:** An overview of the surgical patients enrolled in this study.

Variables	All	Colorectal	Hip Elective	Hip Fracture	*P*
Number of Subjects	30	11	10	9	—
Age	77.9 ± 6.4	73.6 ± 5.8	79.4 ± 4.6	81.3 ± 6.4	1.24E-02
Sex (M:F)	13: 17	7: 4	4: 6	2: 7	—
Smoking (Never: Ex: Current)	20: 10: 0	8: 3: 0	5: 5: 0	7: 2: 0	—
Katz index of ADL	11.9 ± 0.5	12 ± 0	12 ± 0	11.7 ± 0.9	3.22E-01
Charlson’s comorbidity score	1.7 ± 1.3	2.0 ± 1.3	1.9 ± 1.2	1.2 ± 1.3	3.69E-01
ASA class (1–2: 3–4)	23: 7	9: 2	8: 2	6: 3	—
Surgery time (hr)	2.2 ± 1.1	3.0 ± 1.0	1.4 ± 0.5	2.0 ± 1.1	1.13E-02
Anaesthesia time (hr)	2.9 ± 1.1	3.7 ± 1.1	2.3 ± 0.7	2.8 ± 1.1	1.62E-03
Time to POD1 sample (hr)	18.1 ± 3.2	17.4 ± 3.3	20.0 ± 2.6	16.9 ± 3.0	6.35E-02
Time to POD4/7 sample (days)	4.8 ± 1.0	5.5 ± 1.2	4.2 ± 0.4	4.6 ± 0.7	4.24E-03

ADL = activity of daily life. ASA = American Society of Anaesthesiologists. Number or average ± standard deviation is shown for each variable.

### Major surgery is associated with acute changes in DNA methylation, primarily at sites annotated to immune system genes

**Figure 1:**
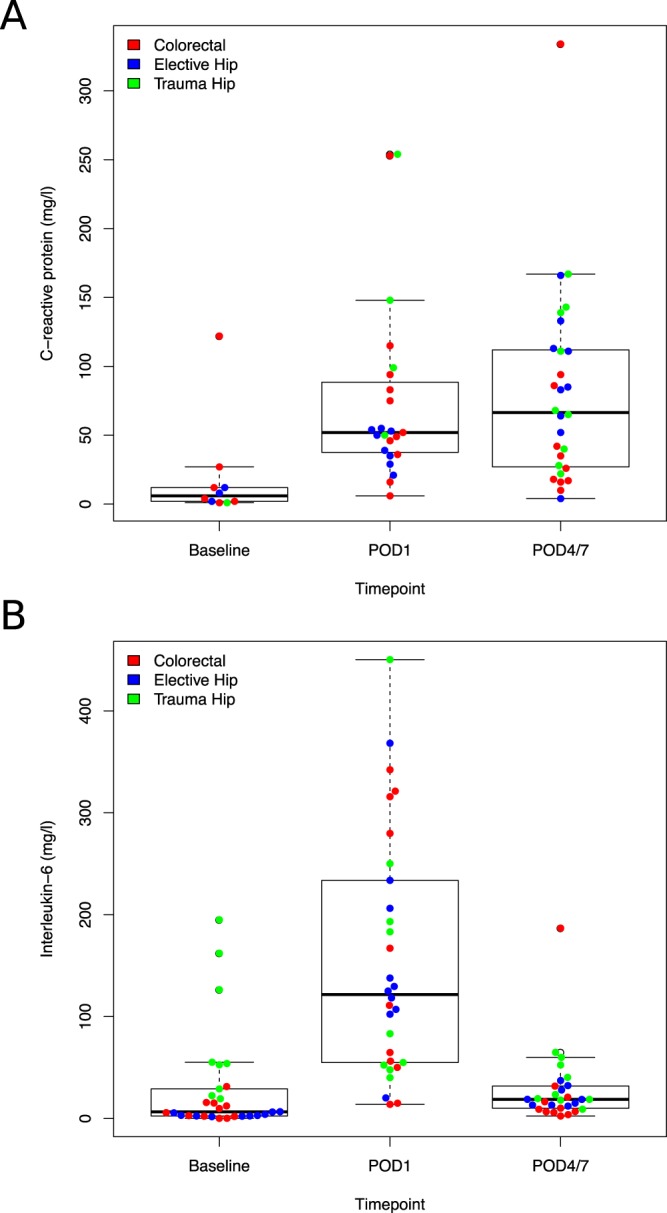
Surgery is associated with acute increases in markers of inflammation. Shown are levels of serum C-reactive protein (CRP) and Interleukin-6 (IL-6) at baseline and after surgery. Both serum CRP (**A**) and IL-6 (**B**) are significantly elevated at POD1 following major surgery (CRP; P = 4.55E-03, IL-6; P = 7.23E-06). Colors denote surgery type: colorectal surgery = red, hip elective surgery = blue, and hip fracture surgery = green.

We first examined acute changes in DNA methylation following major surgery, comparing baseline PBMC samples to those at POD1, collected an average of 18.1 ± 3.2 hours after surgery. In total, we identified 88 DMPs passing an experiment-wide significance threshold (P < 2.0E-07), with 5,655 DMPs passing a more relaxed *“discovery”* significance threshold (P < 5.0E-05) that was used to select genes for subsequent gene ontology (GO) pathway analyses (Supplementary Fig. S2 and Supplementary Table S1). The top ranked DMP (cg15412772), which was significantly hypomethylated following major surgery (DNA methylation change = −4.23%, P = 8.08E-10), is located on chromosome 17 approximately 4 kb downstream of *CSF3*, which encodes a granulocyte colony-stimulating factor that acts to stimulate granulopoiesis and works against neutropenia^15^. We used *comb-p* to identify spatially-correlated regions of differential DNA methylation, identifying 913 surgery-associated DMRs (Supplementary Table S2) (Sidak-corrected P < 0.05)^16^. The top-ranked DMR spans 1,212 bp, incorporating 19 DNA methylation sites on chromosome 6 (P = 2.58E-26), overlapping the transcription start site (TSS) of the gene encoding lymphotoxin-A (*LTA*), which has an important role in regulating the immune system^17^. GO term enrichment analysis on genes annotated to the *“discovery”* DMPs highlighted a highly-significant enrichment for functional pathways associated with immune function amongst surgery-induced DMPs, including categories such as *“immune system process”* (P = 9.51E-24), *“response to wounding”* (P = 1.17E-19) and *“positive regulation of immune response”* (P = 4.50E-12) (Supplementary Table S3). To explore further the acute immune response following major surgery we measured blood serum levels of C-reactive protein (CRP) and Interleukin 6 (IL-6) in the same samples; CRP is an acute phase protein that rises in response to inflammation through hepatic synthesis in response to IL-6, a pro-inflammatory cytokine that is synthesised at the site of injury^18,19^. Compared to baseline we observed a highly significant increase in both serum CRP (baseline: median CRP = 6.0 mg/l; POD1: median CRP = 52 mg/l, P = 4.55E-03) (Fig. 1A) and IL-6 (baseline: median IL-6 = 6.6 pg/ml; POD1: median IL-6 = 121.7 pg/ml, P = 7.23E-06) (Fig. 1B) at POD1. Of note, a recent study described an inflammation-related epigenetic polygenic score based on an analysis of variable DNA methylation associated with CRP in two large population-based cohorts^20^. We applied this predictor to our data, finding a significant increase in the inflammation-related polyepigenetic score at POD1 compared to baseline (P = 2.7E-04, Supplementary Fig. S3), providing further evidence for acute upregulation of the immune system following major surgery. Finally, we assessed whether major surgery is associated with two epigenetic biomarkers of age - the Horvath multi-tissue DNA methylation clock^21^ and the GrimAge clock^22^ that is hypothesized to more accurately index accelerated biological ageing. Estimates from both clocks were associated with actual chronological age at BL (multi-tissue clock: corr = 0.57, P = 5.81E-04; GrimAge clock: corr = 0.79, P = 1.67E-07). Although there was no significant change in estimates derived from the multi-tissue clock following surgery, there was a significant increase in GrimAge detectable one day following surgery (mean change in GrimAge = 1.64 years, P = 2.57E-04, Supplementary Fig. S4), with this elevation being particularly pronounced in patients undergoing acute hip fracture surgery. Of note, previous studies have shown that accelerated GrimAge is associated with inflammatory conditions and an age-related decline in immune system functioning^22^.

### Although major surgery is associated with notable shifts in cell-type proportions, many surgery-associated changes in DNA methylation are independent of blood cell heterogeneity

**Figure 2:**
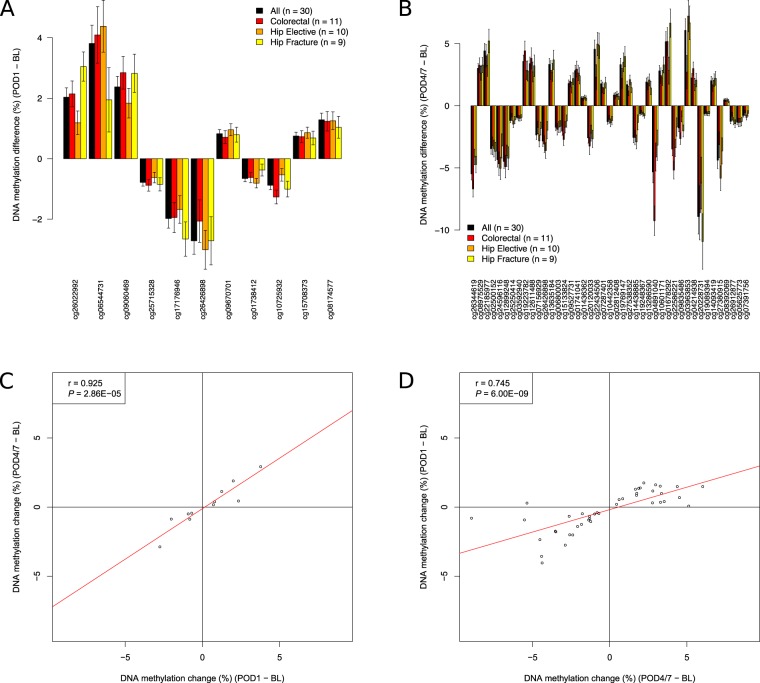
Differentially methylated positions (DMPs) associated with major surgery. (**A**) 11 DMPs passed our experiment-wide significance threshold at POD1 (P < 2.0E-07). Results for each of these sites is given in Table 2. (**B**) 43 DMPs passed our experiment-wide significance threshold at POD4/7 (P < 2.0E-07). Results for each of these sites is given in Table 3. There was a strong correlation of changes observed at POD1 and POD4/7 for DMPs identified at both time-points: (**C**) POD1 DMPs: corr = 0.925, P = 2.86E-05; (**D**) POD4/7 DMPs: corr = 0.745, P = 6.00E-09.

**Figure 3:**
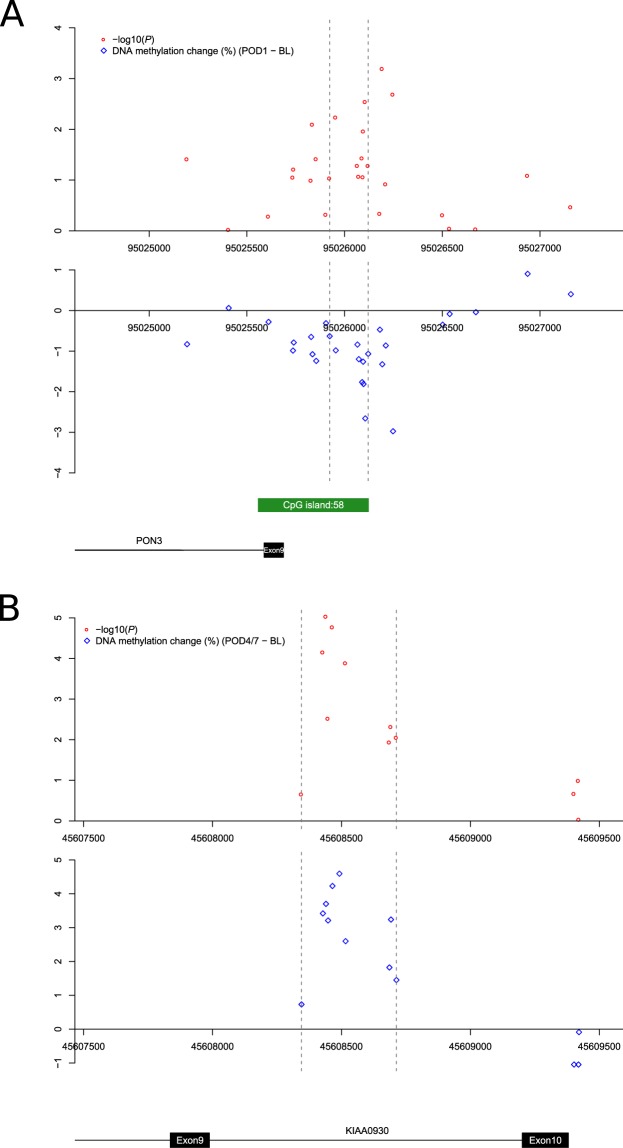
Differentially methylated regions (DMRs) associated with major surgery. (**A**) A DMR on chromosome 7 annotated to the promoter region of PON3 which was hypomethylated (Sidak-corrected P = 1.43E-04) at POD1. (**B**) A DMR on chromosome 22 within KIAA0930 which was hypermethylated (Sidak-corrected P = 3.39E-17) at POD4/7. −log10(P) values and effect sizes (%, average DNA methylation difference) are shown as red circles and blue diamonds, respectively.

Given the highly-significant enrichment of surgery-associated DMPs in the vicinity of genes involved in immune function and cell proliferation, the dramatic surgery-induced changes in CRP and IL-6, and the large observed differences in blood cell proportions and plasma proteins following surgery (Supplementary Figs. S5, S6 and Supplementary Table S4), it is important to control for variable cell populations in our DNA methylation analysis. Although our DNA methylation analyses were performed on PBMC samples depleted for granulocytes, we next used an established deconvolution algorithm to derive blood cell-type proportion estimates from our PBMC DNA methylation data (see Methods), identifying significant acute increases following surgery (between baseline and POD1) in estimates of plasmablasts (P = 2.28E-02) and monocytes (P = 4.43E-05), and significant decreases in naïve CD8 T cells (P = 4.96E-02), CD8 T cells (P = 1.22E-02), CD4 T cells (P = 1.19E-03), and natural killer cells (P = 5.97E-03) (see Supplementary Table S5)^21^. As expected, the estimated level of granulocytes in our isolated PBMCs was very low (mean (±SD) granulocyte proportion = 0.0129 (±0.028)). For the majority of samples we were able to also empirically measure actual blood cell counts; although the specific cell-types derived from DNA methylation data do not overlap entirely with those measured in whole blood, we observed a highly significant correlation between empirically-measured and estimated ratio of lymphocytes and monocytes (r = 0.713, P = 8.53E-15, Supplementary Fig. S7), indicating that the derived cell proportion estimates are likely to reflect actual levels in our samples. These shifts in cell-type proportions are themselves interesting, reflecting known surgery-related immune outcomes; for example previous studies have highlighted a dramatic increase and upregulation of monocytes in the inflammatory response following surgery^23^ with lymphocytes decreasing in a cell-subtype specific manner^24^. Because such cellular changes might confound our DNA methylation analyses, we used a multi-level regression model including the estimated proportions of plasmablasts, CD8 + CD28-CD45RA- T cells, naive CD8 T cells, CD4T cells, natural killer cells and monocytes for each sample (derived from the DNA methylation data), in addition to age and sex, to further explore acute surgery-associated DMPs between baseline and POD1 (see Methods). Although there was a strong correlation for both effect sizes (r = 0.955, P = 2.20E-47, Supplementary Fig. S8) and P values (r = 0.621, P = 6.45E-11, Supplementary Fig. S9) between models for DMPs identified in our uncorrected model, the cell-proportion-corrected model identified a smaller number of significant surgery-associated changes. In total, we identified 11 DMPs passing an experiment-wide significance threshold P < 2.0E-07 (Fig. 2A and Table 2) and 475 DMPs passing a more relaxed *“discovery”* significance threshold (P < 5.0E-05) (Supplementary Fig. S10 and Supplementary Table S6). The top ranked DMP (cg26022992), which was significantly hypermethylated following major surgery (DNA methylation change = 2.04%, P = 1.02E-08), is located on chromosome 1 in the 5′ untranslated region of *RBM8A*, a gene encoding a protein with a conserved RNA-binding motif that is involved in splicing. We again used *comb-p*^16^ to identify spatially-correlated regions of differential DNA methylation, with the top-ranked DMR spanning nine hypomethylated sites on chromosome 7 immediately upstream of *PON3*, a member of the paraoxonase family that associates with high-density lipoprotein and has anti-inflammatory and anti-oxidant properties^25^ (Sidak-corrected P = 1.43E-4) (Fig. 3A and Supplementary Table S7). GO term enrichment analysis of genes annotated to the list of ‘*discovery*’ DMPs again highlighted a significant enrichment for functional pathways associated with immune function amongst surgery-induced DMPs, for example *“response to interleukin-15”* (P = 3.44E-10), in addition to some more physiological pathways including *“regulation of muscle adaptation”* (P = 5.23E-06) and *“respiratory system process”* (P = 5.91E-05) (Supplementary Table S8). Finally, we explored whether within-individual changes in DNA methylation were associated with shifts in serum IL-6 levels following major surgery. We identified 43 sites at which altered DNA methylation was associated with the magnitude of change in serum IL-6 levels (P < 2.0E-07, Supplementary Table S9), with the top-ranked site (cg24327971) annotated to *MRPL3*, a mitochondrial ribosomal protein located on chromosome 3 (DNA methylation change per 10 pg/ml IL-6 = 0.15%, P = 4.32E-10).

**Table 2:** Sites characterized by significant changes in DNA methylation on post-operative day 1 compared to baseline.

DMP	Position	GREAT annotation	Surgery type											
			All (n = 30)			Colorectal (n = 11)			Hip Elective (n = 10)			Hip Fracture (n = 9)		
			BL DNAm (%)	DNAm change (%)	*P* value	BL DNAm (%)	DNAm change (%)	*P* value	BL DNAm (%)	DNAm change (%)	*P* value	BL DNAm (%)	DNAm change (%)	*P* value
cg26022992	chr1:145507616	RBM8A (+60)	8.24	2.04	1.02E-08	8.21	1.19	3.45E-03	8.62	2.14	4.68E-06	7.87	3.05	3.69E-08
cg06544731	chr1:24738219	NIPAL3 (−4025)	67.97	3.81	3.50E-08	66.68	4.38	3.54E-06	68.45	4.09	5.03E-05	69.01	1.95	7.19E-02
cg09060469	chr1:204912595	CNTN2 (−99744), NFASC (+114814)	78.73	2.38	3.66E-08	79.29	1.84	6.90E-04	78.38	2.85	7.29E-06	78.42	2.82	5.64E-05
cg25715328	chr19:9649297	ZNF426 (+5)	4.52	−0.78	4.49E-08	4.86	−0.62	7.12E-04	4.23	−0.88	2.12E-05	4.43	−0.85	2.30E-04
cg17176946	chr11:108811287	C11orf87 (−481558), DDX10 (+275472)	87.11	−1.98	5.13E-08	87.00	−1.68	5.35E-04	87.13	−1.94	2.60E-04	87.20	−2.66	1.68E-05
cg26426898	chr20:709094	SCRT2 (−52272), C20orf54 (+40133)	40.41	−2.72	7.15E-08	41.28	−3.01	1.63E-05	39.28	−2.06	4.65E-03	40.60	−2.71	1.17E-03
cg09670701	chr15:34876396	GOLGA8B (−48175), GJD2 (+170292)	5.72	0.83	8.19E-08	5.68	0.96	9.40E-06	5.72	0.71	1.72E-03	5.78	0.79	1.94E-03
cg01738412	chr7:87505461	DBF4 (−82), SLC25A40 (+230)	4.94	−0.66	1.26E-07	5.20	−0.81	3.52E-06	4.84	−0.62	6.70E-04	4.74	−0.37	6.10E-02
cg10725932	chr11:71934276	INPPL1 (−1605)	5.94	−0.88	1.27E-07	5.72	−0.53	1.29E-02	6.05	−1.27	6.36E-07	6.10	−1.00	2.53E-04
cg15708373	chr8:81397789	ZBTB10 (−658)	4.42	0.75	1.39E-07	4.36	0.86	1.31E-05	4.39	0.73	5.08E-04	4.54	0.69	3.42E-03
cg08174577	chr2:52007582	NRXN1 (−747909)	87.34	1.29	1.60E-07	87.39	1.26	6.86E-05	87.60	1.24	2.78E-04	86.98	1.04	5.79E-03

Shown for each DMP are the genomic location (hg19) and genic annotation derived using GREAT42. Association statistics are presented across all patients, and separately for each surgery type. DMP = differentially methylated positions. BL = baseline. DNAm = DNA methylation.

### Surgery-associated shifts in DNA methylation regulating the immune response are relatively stable until discharge from hospital

We next extended our analysis to characterize more long-term changes in immune response markers and DNA methylation following major surgery (i.e. those occurring between BL and POD4/7), exploring the stability of acute surgery-induced changes identified at POD1. Compared to baseline we again observed a highly significant increase in serum CRP at POD4/7 (baseline: median CRP = 6.0 mg/l; POD4/7: median CRP = 66.5 mg/l, P = 1.39E-03) (Fig. 1A), with no significant change between POD1 and POD4/7 (P = 7.25E-01). We also found a significant increase in the inflammation-related polyepigenetic score derived from our DNA methylation data^20^ at POD4/7 compared to baseline (P = 3.3E-4, Supplementary Fig. S3), again with no significant change between POD1 and POD4/7 (P = 6.54E-01). In contrast, after peaking at POD1, levels of IL-6 returned back to baseline levels by POD4/7; interestingly IL-6 is known to drive the initial inflammatory response, increasing CRP via interaction with STAT^26^ before rapidly returning back to normal levels. Over the perioperative period (including samples collected at BL, POD1, and POD4/7) both serum CRP (corr = 0.32, P = 7.16E-03) and IL-6 (corr = 0.23, P = 1.84E-02) were found to be positively correlated with the inflammation-related polyepigenetic score (Supplementary Fig. S3), although the changes in this predictor more closely reflect CRP than IL-6. In total we identified 43 DMPs characterized by an experiment-wide significant difference (P < 2.0E-07) at POD4/7 compared to BL using a model correcting for derived blood cell-type proportions (Fig. 2B and Table 3), with 909 DMPs identified at a more relaxed “discovery” significance threshold (P < 5.0E-05) (Supplementary Fig. S11 and Supplementary Table S10). The top-ranked DMP at POD4/7 (cg26344619), which is significantly hypomethylated following surgery (DNA methylation change = −5.49%, P = 1.13E-13), is located within the first intron of *FLVCR2* on chromosome 14, a gene encoding a transmembrane protein involved in heme and calcium transport^27,28^. The top-ranked DMR at POD4/7 spans ten hypermethylated sites on chromosome 22 in the promoter region of *KIAA0930* (Sidak-corrected P = 3.39E-17) (Fig. 3B and Supplementary Table S7). Functional pathways enriched amongst genes annotated to DMPs at POD4/7 again included immune response pathways such as “*cellular response to interleukin-6*” (P = 3.31E-06) in addition to several cytoskeletal pathways including “*actomyosin*” (P = 6.09E-06) and “*filamentous actin*” (P = 7.43E-06), which play an active role in wound healing and tissue regeneration^29,30^ (Supplementary Table S11). It is interesting that more DMPs were identified at POD4/7 than at POD1; although DNA methylation at only one site (cg26426898 on chromosome 20, annotated to *SCRT2* and *C20orf54*) was identified as significantly different (P < 2.0E-07) at both POD1 and POD4/7 compared to BL, there was a strong correlation of changes observed at POD1 and POD4/7 for DMPs identified at both time-points (POD1 DMPs: corr = 0.925, P = 2.86E-05, Fig. 2C; POD4/7 DMPs: corr = 0.745, P = 6.00E-09, Fig. 2D) indicating that surgery-induced DNA methylation changes are relatively stable until the time of discharge. Together, our results support the notion that the immune-related epigenetic changes associated with major surgery occur rapidly and are relatively stable across the post-operative period.

**Table 3:** Sites characterized by significant changes in DNA methylation on post-operative day 4 to 7 compared to baseline.

DMPs	Position	GREAT annotation	Surgery type											
			All (n = 30)			Colorectal (n = 11)			Hip Elective (n = 10)			Hip Fracture (n = 9)		
			BL DNAm (%)	DNAm change (%)	*P* value	BL DNAm (%)	DNAm change (%)	*P* value	BL DNAm (%)	DNAm change (%)	*P* value	BL DNAm (%)	DNAm change (%)	*P* value
cg26344619	chr14:76046018	FLVCR2 (+1079), C14orf1 (+81519)	59.43	−5.49	1.13E-13	58.95	−4.12	3.12E-07	61.69	−6.69	1.07E-10	57.52	−4.73	1.12E-07
cg08975529	chr11:70309722	CTTN (+65111), SHANK2 (+626119)	79.53	3.01	4.19E-10	79.20	2.62	4.05E-05	80.02	3.26	3.55E-06	79.39	3.28	4.85E-06
cg22185977	chr5:1518133	SLC6A3 (−72591), LPCAT1 (+5942)	57.93	4.42	5.21E-10	57.48	3.24	4.20E-04	61.17	4.02	4.86E-05	54.89	5.22	7.18E-07
cg02500152	chr12:48357316	TMEM106C (−13)	20.07	−3.46	8.13E-10	20.78	−3.44	1.11E-05	19.04	−3.23	6.50E-05	20.35	−3.67	1.43E-05
cg24596116	chr12:68665317	IL22 (−18037), MDM1 (+60843)	61.35	−4.34	9.30E-10	61.97	−5.04	5.22E-07	61.51	−4.67	6.90E-06	60.42	−3.24	1.48E-03
cg12899248	chr17:15845357	ADORA2B (−2873)	81.72	−4.48	1.14E-09	81.54	−4.02	3.68E-05	82.07	−4.90	4.35E-06	81.56	−4.22	5.87E-05
cg25250414	chr15:75871478	PTPN9 (+146)	7.18	−1.19	2.81E-09	7.13	−1.48	5.64E-07	6.94	−0.90	2.15E-03	7.53	−1.14	1.93E-04
cg03592940	chr2:74153896	DGUOK (−56)	5.42	−0.94	5.57E-09	5.35	−1.02	1.29E-05	5.29	−0.99	4.66E-05	5.65	−0.95	1.34E-04
cg19223782	chr11:503807	RNH1 (+3013), PTDSS2 (+53528)	66.64	3.57	1.51E-08	66.71	2.85	5.22E-04	68.21	4.42	2.87E-06	64.82	2.80	1.51E-03
cg12611488	chr1:2169282	RER1 (−153931), SKI (+9149)	82.79	3.40	1.64E-08	83.20	2.72	1.21E-03	83.08	3.82	3.04E-05	81.98	3.22	4.53E-04
cg07156929	chr6:34856927	TAF11 (−1109), ANKS1A (−110)	21.55	−2.35	1.94E-08	22.91	−2.83	1.71E-07	20.28	−1.75	9.82E-04	21.29	−1.83	8.04E-04
cg26426898	chr20:709094	SCRT2 (−52272), C20orf54 (+40133)	40.41	−2.84	2.00E-08	41.28	−3.61	4.45E-07	39.28	−3.09	2.37E-05	40.60	−1.97	6.06E-03
cg13635184	chr7:148903126	ZNF212 (−33615), ZNF282 (+10550)	83.84	3.33	2.46E-08	83.26	2.68	6.94E-04	84.11	2.86	6.24E-04	84.24	3.69	3.59E-05
cg00680003	chr6:30523449	PRR3 (−1036)	14.31	−1.74	3.08E-08	14.85	−1.72	1.91E-04	14.12	−1.90	1.03E-04	13.86	−1.66	7.75E-04
cg15133824	chr2:97523823	ANKRD23 (−14066), SEMA4C (+11911)	18.61	−2.04	3.20E-08	18.57	−1.73	3.56E-04	18.72	−2.71	5.00E-07	18.52	−1.23	1.58E-02
cg09527731	chr5:132097228	CCNI2 (+14092), SEPT8 (+15838)	88.08	1.79	3.27E-08	88.10	1.46	1.18E-03	88.26	1.97	5.18E-05	87.86	2.19	1.54E-05
cg01741041	chr7:5567862	FBXL18 (−14464), ACTB (+2369)	20.45	2.83	3.89E-08	21.45	2.64	1.32E-04	18.97	2.95	5.69E-05	20.87	2.41	8.10E-04
cg01436362	chr1:229479015	C1orf96 (−328)	4.28	0.65	4.72E-08	4.26	0.70	6.92E-05	4.28	0.59	1.02E-03	4.33	0.62	8.44E-04
cg20120033	chr3:65367695	ADAMTS9 (−694331), MAGI1 (+656813)	83.51	−2.59	5.02E-08	83.31	−2.20	1.16E-03	83.78	−3.25	1.22E-05	83.46	−2.66	3.32E-04
cg22434506	chr12:6657818	IFFO1 (+7430), GAPDH (+14162)	81.19	4.56	5.08E-08	79.68	4.96	1.74E-06	84.11	2.31	2.32E-02	79.78	4.84	1.25E-05
cg07287401	chr10:134054776	DPYSL4 (+54363), STK32C (+66700)	87.43	1.77	5.92E-08	86.73	1.36	1.22E-03	87.91	1.48	9.06E-04	87.74	1.86	7.02E-05
cg10442358	chr7:100137102	AGFG2 (+269)	7.44	−1.28	6.11E-08	7.76	−1.37	5.07E-05	7.03	−1.10	1.61E-03	7.51	−1.19	8.75E-04
cg02812408	chr6:25153566	LRRC16A (−126089), CMAHP (−15516)	92.99	0.88	6.32E-08	92.88	0.94	7.45E-05	93.07	0.83	7.69E-04	93.05	0.75	2.63E-03
cg19769147	chr14:105860954	TEX22 (−3965)	54.64	3.31	6.64E-08	54.92	3.49	4.20E-05	55.71	2.21	8.38E-03	53.10	3.96	2.11E-05
cg27538352	chrX:49032410	PLP2 (+4227), PRICKLE3 (+10365)	90.71	1.71	8.33E-08	89.98	2.10	4.40E-07	91.47	1.20	3.42E-03	90.76	1.46	6.11E-04
cg14438885	chr17:59475396	TBX2 (−1860)	20.04	−2.55	8.45E-08	20.98	−2.91	2.14E-06	19.28	−2.55	5.29E-05	19.73	−1.34	3.00E-02
cg19248367	chr11:67141079	CLCF1 (+568)	4.03	−0.66	8.56E-08	4.03	−0.67	1.35E-04	3.84	−0.54	2.97E-03	4.25	−0.82	2.50E-05
cg13286590	chr6:31778018	LSM2 (−3258)	82.11	1.92	9.92E-08	81.82	2.07	6.05E-05	82.15	1.81	7.41E-04	82.42	1.43	8.02E-03
cg04891040	chr16:66399929	CDH5 (−595)	58.61	−5.32	1.03E-07	59.08	−4.12	8.82E-04	61.18	−9.26	7.98E-09	55.17	−3.17	1.39E-02
cg10601171	chr20:61290162	NTSR1 (−50026), SLCO4A1 (+16366)	66.38	2.81	1.12E-07	67.58	2.24	7.95E-04	65.59	2.43	4.00E-04	65.81	3.29	1.12E-05
cg01678292	chr8:41522873	NKX6-3 (−17999), ANK1 (+231406)	59.07	5.16	1.16E-07	59.69	2.83	1.31E-02	59.87	5.17	5.18E-05	57.41	6.65	1.43E-06
cg22586221	chr15:78984179	CHRNB4 (−50593), ADAMTS7 (+119593)	61.25	−3.49	1.18E-07	62.28	−3.38	2.91E-05	61.30	−5.18	8.74E-08	59.93	−1.71	2.70E-02
cg09835486	chr5:112257968	REEP5 (+62)	11.10	−1.79	1.20E-07	10.85	−0.85	5.72E-02	11.21	−2.64	4.30E-07	11.29	−2.02	7.91E-05
cg03963853	chr16:4732369	NUDT16L1 (−11324), MGRN1 (+57545)	72.46	6.07	1.23E-07	71.96	7.22	1.89E-06	75.66	2.71	4.95E-02	69.52	6.67	2.34E-05
cg04214938	chr2:119496171	EN1 (+109587), INSIG2 (+650122)	85.04	2.25	1.24E-07	85.62	1.71	1.81E-03	84.88	2.99	4.16E-06	84.51	2.07	6.76E-04
cg20228731	chr7:130646051	MKLN1 (−366543), KLF14 (−227164)	33.17	−8.92	1.24E-07	30.78	−6.22	4.92E-03	33.54	−8.61	3.50E-04	35.66	−10.93	1.97E-05
cg19089394	chr11:66610747	RCE1 (−135)	3.17	−0.66	1.33E-07	3.36	−0.65	1.90E-04	2.93	−0.59	1.04E-03	3.21	−0.64	6.47E-04
cg14093419	chr7:152591099	ACTR3B (+134249)	86.21	2.02	1.45E-07	86.24	2.06	1.55E-04	86.82	1.69	2.71E-03	85.51	2.21	1.84E-04
cg27380915	chr7:12796513	ARL4A (+70062)	68.30	−4.38	1.46E-07	68.06	−5.83	9.09E-07	70.81	−3.13	4.50E-03	65.82	−3.64	1.46E-03
cg08392069	chr16:2205170	TRAF7 (−628)	3.07	0.48	1.57E-07	2.98	0.49	1.58E-04	3.21	0.45	9.61E-04	3.04	0.38	5.18E-03
cg26912877	chr1:6305344	HES3 (+1093), GPR153 (+15690)	7.20	−1.26	1.57E-07	7.57	−1.24	1.46E-04	6.83	−0.85	9.37E-03	7.17	−1.45	6.13E-05
cg05625773	chr6:30614796	C6orf136 (−19)	8.74	−1.32	1.84E-07	8.90	−1.34	2.15E-04	8.34	−1.35	3.81E-04	8.97	−1.28	9.41E-04
cg07391756	chr12:132568647	DDX51 (+60232), EP400 (+134183)	5.75	−0.78	1.84E-07	6.27	−0.94	1.17E-05	5.43	−0.57	8.43E-03	5.48	−0.61	5.78E-03

Shown for each DMP are the genomic location (hg19) and genic annotation derived using GREAT^42^. Association statistics are presented across all patients, and separately for each surgery type. DMP = differentially methylated positions. BL = baseline. DNAm = DNA methylation.

### Surgery-associated DNA methylation differences identified by the Illumina 450 K were robustly validated using bisulfite-pyrosequencing

We used bisulfite-pyrosequencing to confirm surgery-associated changes in DNA methylation at several DMPs, validating the results of our Illumina 450 K analyses and finding highly consistent results across platforms. First, we re-quantified DNA methylation at cg24501381, one of the top-ranked DMPs from our initial analysis uncorrected for cell-types (average DNA methylation change = −4.99%, P = 5.69E-09) that is annotated to *CCDC30* on chromosome 1. There was a highly significant correlation between Illumina 450 K array and bisulfite-pyrosequencing datasets (P = 1.04E-23, Supplementary Fig. S12), with the pyrosequencing data confirming the surgery-associated reduction in DNA methylation (average DNA methylation change = −4.37%, P = 8.75E-06, Supplementary Fig. S13). Second, we re-quantified DNA methylation at cg26344619, the top-ranked DMP at POD4/7, again finding a highly significant correlation between 450 K array and bisulfite-pyrosequencing datasets (P = 5.38E-19, Supplementary Fig. S14), with the pyrosequencing data confirming the surgery-associated reduction in DNA methylation (average DNA methylation change = −4.90%, P = 7.07E-05, Supplementary Fig. S15).

### Heterogeneity in DNA methylation changes between different types of surgery

The DNA methylation changes at POD1 and POD4/7 described above are relatively consistent across the three types of major surgery (colorectal elective surgery, hip elective surgery, and hip fracture surgery) (Fig. 2A,B). It is, however, possible that there are unique effects specific to individual surgery-type groups or between emergency and elective surgery. The three groups were relatively well-matched for most demographic and surgical parameters; although there were no differences in smoking status, marital status, Charlson’s comorbidity score, Katz index, American Society of Anesthesiologists (ASA) classification, or blood loss between surgery types, patients undergoing colorectal surgery were younger (P = 1.24E-02) and were exposed to a longer duration of surgery (P = 1.13E-02) than the other groups (Table 1). Anaesthesia time was also longer in patients in the colorectal surgery group than those undergoing hip elective surgery (P = 1.62E-03). We used an analysis model designed to identify changes in DNA methylation that differed between surgery types (see Methods), finding eight surgery-type-specific DMPs (P < 2.0E-07) at POD1 (Supplementary Table S12 and Supplementary Fig. S16) and eleven at POD4/7 (Supplementary Table S13 and Supplementary Fig. S17). Given the relatively small numbers of patients in each of the individual surgery groups, however, these stratified analyses are relatively underpowered and should be considered with some caution.

## Discussion

In this study we characterized longitudinal changes in DNA methylation in peripheral blood PBMCs collected from elderly patients undergoing major surgery. Our primary analyses focused on comparing samples collected at baseline to those collected immediately post-operatively and at discharge from hospital. We observed rapid changes in DNA methylation following surgery, with widespread differences becoming manifest before the morning of postoperative day 1. The acute changes in DNA methylation induced by surgery are relatively stable in the post-operative period, generally persisting until discharge from hospital.

We found that major surgery is associated with acute changes in DNA methylation at sites annotated to immune system genes, reflecting the observed changes in serum-levels of markers associated with surgical trauma, including CRP and IL-6, in the same samples. Of note, previous studies have identified changes in DNA methylation associated with inflammation and the immune response^31,32^. This physiological response involves dynamic changes in gene regulation, and the acute shifts in DNA methylation observed in our study parallel the immunological responses to major surgery that underlie the post-operative recovery^7^ and which have been associated with susceptibility to certain postoperative complications such as nosocomial infection, delirium, and cognitive dysfunction^4,5^. Given the growing need to optimise surgical recovery in our aging population, our findings highlight how perioperative molecular approaches might provide insight into the pathophysiology of surgical complications, and future work will focus on stratifying individuals to identify methylomic signatures of poor outcome^33^. Although many of the observed changes in DNA methylation are consistent across the three types of surgery, there is notable heterogeneity between surgery types at certain loci. These differences may reflect different types of surgical procedures, emergency and elective surgery, or even the tissues operated upon, the surgical access type and underlying pathology.

This study has several strengths and weaknesses that should be considered when interpreting the results of our analyses. First, we implemented a prospective study design, longitudinally sampling individuals before and at several time-points after major surgery. The analysis of sequential samples from the same individual that negates many of the important confounds that can influence molecular epidemiology as we are using each patient as their own baseline compared to an external reference level from a heterogeneous population and we can control for variables such as age, sex and exposure (e.g. to smoking and medication)^34^. Second, although we isolated PBMCs from fresh whole blood, cellular heterogeneity remains an important potential confounder especially given the known blood-cell changes known to occur following surgery. Of note, we were able to correct for blood cell proportions, albeit only for major cell-types, and show that our derived estimates reflected directly measured cell-type levels. Our results support the usefulness of cell-correction algorithms in epigenetic epidemiology and highlight the importance of adjusting for cell type composition for the epigenetic research in perioperative period. Despite this, it is possible that our results reflect shifts in subpopulations of PBMCs as well as by activation of specific cell subsets. Furthermore, given the damage to other tissues that invariable take place during surgery, it is possible that our results are also confounded by DNA and cellular components from other organs. Third, we quantified DNA methylation using the Illumina 450 K array; although this platform interrogates sites annotated to the majority of genes, the actual proportion of sites across the genome interrogated by this technology is relatively low, with a predominant focus on Cytosine-guanine dinucleotide (CpG) rich promoter regions. It will be important for future studies to explore surgery-induced changes in DNA methylation across regions not well-covered by the Illumina 450 K array. Of note, we used bisulfite-Pyrosequencing to validate surgery-induced DNA methylation changes at several loci, confirming the robustness of the 450 K array for identifying real DNA methylation changes. Fourth, we did not assess changes in gene expression resulting from the methylomic variation induced by surgery, and this should be a focus of future work. However, we did find dramatic changes in both CRP and IL-6 levels in serum from the same individuals using ELISA, confirming functional effects on the immune system. Although the magnitude of changes in DNA methylation observed were relatively small, they are statistically robust and occur at sites annotated to genes with a known role in the immunological response. Finally, the inclusion of patients undergoing multiple surgery types enabled us to explore the consistency of DNA methylation changes following major surgery. Despite this, however, individual surgery-type groups were relatively small and future work is needed to further interrogate genomic changes specific to each group. Surgery is a complex procedure and involves a large combination of factors that could influence DNA methylation including the surgical approach (open versus minimally-invasive), differences in anaesthetic techniques used, differences in the tissues undergoing surgery, and different rates of complications and compounding factors. Future work in larger surgical cohorts will enable us to address these factors.

To our knowledge, this represents the first study to systematically explore acute changes in gene regulation following major surgery. We identified acute surgery-associated changes in DNA methylation which are relatively stable until discharge from hospital. Taken together, our results highlight the dramatic alterations in gene regulation induced by invasive surgery, primarily reflecting upregulation of the immune system in response to trauma.

## Methods

### Participants and sample collection

Potential participants were identified by clinicians during routine clinical practice. Between July 2014 and January 2015, a total of 55 patients undergoing routine/emergency surgery at the Royal Devon & Exeter Hospital (Exeter, UK) were recruited via the Royal Devon and Exeter Tissue Bank (RDETB). The ethically approved RDETB (REC ref: 11/SW/0018) was set up to proactively collect and store tissue available from routine clinical procedures for studies examining disease specific biomarkers and is facilitated through the NIHR Exeter Clinical Research Facility (ECRF) https://exetercrfnihr.org/about/rde-tissue-bank/. This study was conducted in accordance with the 1964 Helsinki Declaration. Patients with known dementia, cognitive decline (defined as <9/10 points on the Abbreviated Mental Test^35^), delirium before surgery, early discharge within 3 days after surgery were excluded from participating in the study. Written informed consent was obtained from all participants. Pre-operatively, patients were assessed using the Katz index^36^, the Charlson’s comorbidity score^37^ and the ASA classification, with the absence of delirium was assessed using the Confusion Assessment Method^38^. Peripheral whole blood samples were obtained at three time points: 1) immediately before surgery at baseline (“BL”), 2) in the morning on post-operation day 1 (“POD1”) and 3) on day 4–7 immediately prior to discharge (“POD4/7”). Whole blood was taken by a trained research nurse in addition to clinical examination and samples were immediately transferred to the research team for isolation of PBMCs. Out of the 55 patients recruited to the study, a complete set of biological and clinical samples were collected from 30 patients. A number of patients were excluded; unstable/severe clinical condition (n = 2), cancelled surgery (n = 4), failed sample collection (n = 2), history of delirium (n = 2), or early discharge and a failure to collect three blood samples (n = 15). An overview of the patients included in our final analysis dataset is given in Supplementary Fig. S1.

### IL-6, CRP, and blood component measurements

Serum was collected from fresh blood samples by centrifugation at 1,600 × g at room temperature for 20 minutes and stored at −80 °C until use. Serum IL-6 levels were measured using the Quantikine Enzyme Linked Immuno-Sorbent Assay Human IL-6 Kit (R&D Systems, Minneapolis, Minnesota, United States) following the manufacturers standard protocol. Each experiment was performed in duplicate and calibrated using standards. Serum levels of CRP and other blood components were measured in the hospital clinical chemistry laboratory. Laboratory-derived data measured within 3 days before surgery were accepted as baseline.

### Genome-wide DNA methylation profiling

PBMCs were isolated from fresh whole blood, 15 minutes to 2 hours after collection, using Vacutainer CPT Mononuclear Cell Preparation Tubes (Becton Dickinson, Franklin Lakes, New Jersey, United States). Ficoll-separated PBMCs were centrifuged at 1,600 × g at room temperature for 20 minutes, and then washed twice with 5 ml PBS. Isolated PBMCs were resuspended in RNAprotect cell reagent (Qiagen, Redwood City, California, United States) and stored at −80 °C. Genomic DNA was extracted using the AllPrep DNA/RNA minikit (Qiagen) and bisulfite converted using the EZ-96 DNA Methylation-Gold kit (Zymo research, Irvine, California, United States) following the manufacture’s standard protocol. Aliquoted bisulfite converted DNA was amplified with bisulfite specific primers to confirm conversion efficiency prior to array analysis. Genome-wide DNA methylation was profiled using the Illumina Infinium HumanMethylation450 BeadChip (Illumina Inc, San Diego, California, United States) (“450 K array”) and scanned on an Illumina iScan. Our stringent quality control pipeline included the following steps: (1) checking methylated and unmethylated signal intensities; (2) using the 10 control probes to ensure the bisulfite conversion was successful, excluding any samples with median <90; (3) multidimensional scaling of sites on X and Y chromosomes separately to confirm reported gender; (4) comparison of genotype data for up to 65 single nucleotide polymorphism (SNP) probes on 450 K array; and (5) use of the pfilter() function from the *wateRmelon* package to exclude samples with >1% of probes with detection P value > 0.05 and probes with >1% of samples with detection P value > 0.05. The data was subsequently normalized using the *dasen* function in the *wateRmelon* package for a combination of background adjustment and between-array quantile normalization^39^. Probes with non-specific sequences and those containing common (Minor allele frequency >5%) single nucleotide polymorphism probes in European population were removed^40^ leaving 427,353 probes for analysis.

### Bisulfite-pyrosequencing

Bisulfite-pyrosequencing was performed to validate DNA methylation data from the Illumina 450 K array for two CpG sites using specific primers (Supplementary Table S14) designed using PyroMark Assay Design software 2.0 (Qiagen). DNA methylation was quantified from intensities captured in the PyroMark Q24 pyrosequencer (Qiagen) based on duplicated bisulfite polymerase chain reaction amplification.

### Statistical analyses

All statistical analyses were performed in the R statistical environment (version, 3.3.2). Surgery-induced DNA methylation across the genome was first examined using paired t-test for 427,353 DNA methylation sites included in our dataset following QC. We then used a multi-level linear regression model fitted for each DNA methylation site with fixed effects for estimated cell counts (derived using the Epigenetic Clock Software^21^: https://labs.genetics.ucla.edu/horvath/dnamage/), age, sex, and processing batch and a random effect for individual using the *lme4* R package^41^. For Illumina array analyses we used a threshold of P < 2.0E-7 for genome wide significance and P < 5.0E-05 to select probes for GO pathway analyses. Demographic data were tested using analysis of variance (ANOVA). Laboratory-derived blood data alterations following surgery were tested using a paired t-test. CRP levels were assessed using a two-tailed t-test because of decreased sample size in sequential measures.

### Ethics approval and consent to participate

This study was approved by the RDETB Steering Committee under the terms of the overall RDETB ethical approval (REC ref: 11/SW/0018). All participants provided written informed consent for participation. This project was conducted in accordance with the 1964 Helsinki Declaration.

## Supplementary information

Supplementary Tables

Supplementary Figures

Supplementary Table Legends

## Data Availability

Raw data are available to download from the Gene Expression Omnibus (GEO, https://www.ncbi.nlm.nih.gov/geo/) and analysis scripts are available from the authors on request.
