# Supplementary material for: Major surgery induces acute changes in measured DNA methylation associated with immune response pathways
Source: Sci Rep. 2020 Apr 1;10:5743. doi: 10.1038/s41598-020-62262-x (PMC7113299; doi:10.1038/s41598-020-62262-x)

SUPPLEMENTARY FIGURES

Major surgery induces acute changes in measured DNA methylation associated with immune response pathways.

Ryoichi Sadahiro[1,2,*], Bridget Knight[2,3], Ffion James[2], Eilis Hannon[2], John Charity[3], Ian R. Daniels[3], Joe Burrage[2], Olivia Knox[2], Bethany Crawford[2], Neil J. Smart[3], Jonathan Mill[2*]

[1] Department of Immune Medicine, National Cancer Center Research Institute, National Cancer Center Japan, Japan
[2] University of Exeter Medical School, University of Exeter, United Kingdom
[3] Royal Devon & Exeter Hospital, Exeter, United Kingdom

Supplementary Figure S1 – An overview of the study design. We recruited a cohort of 30 elderly patients (average age = 77.9 years) undergoing major surgery (colorectal elective surgery (n = 11), elective hip replacement surgery (n = 10), and hip fracture surgery (n = 9)). From each individual, detailed blood measures were collected and DNA methylation was profiled using the Illumina HumanMethylation450 array in DNA samples isolated from peripheral blood mononuclear cells (PBMCs) collected at three time-points: i) immediately before surgery (baseline), ii) in the morning of post-operative day 1 (POD1) and iii) on post-operative day 4 to 7 (POD4/7) before discharge from hospital.

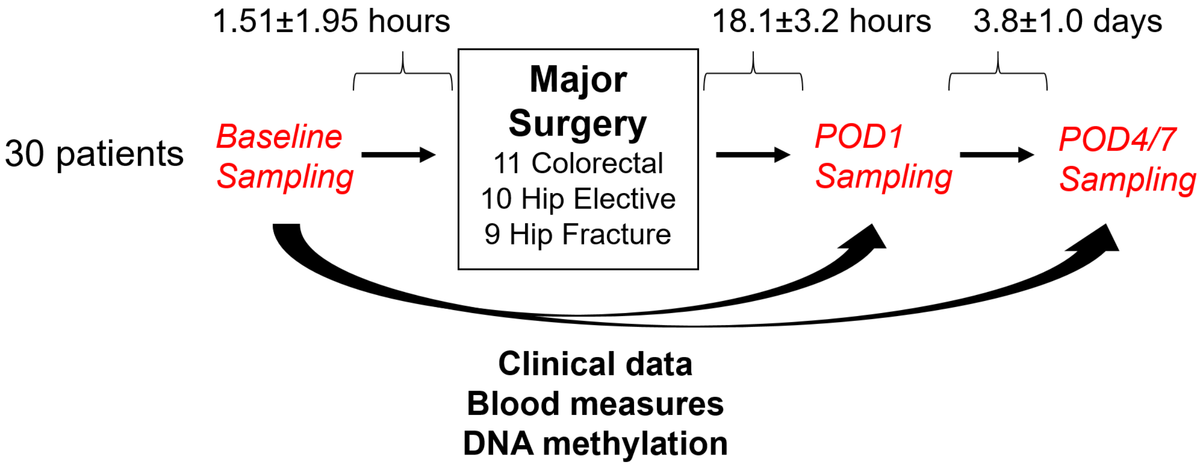

Supplementary Figure S2 – Manhattan plot for acute DNA methylation changes associated with major surgery. Within each individual, DNA methylation was compared between BL and POD1. Red line = experiment-wide significance threshold (P < 2.0E-07). Blue line = discovery significance threshold (P < 5.0E-05) used for gene ontology analysis.

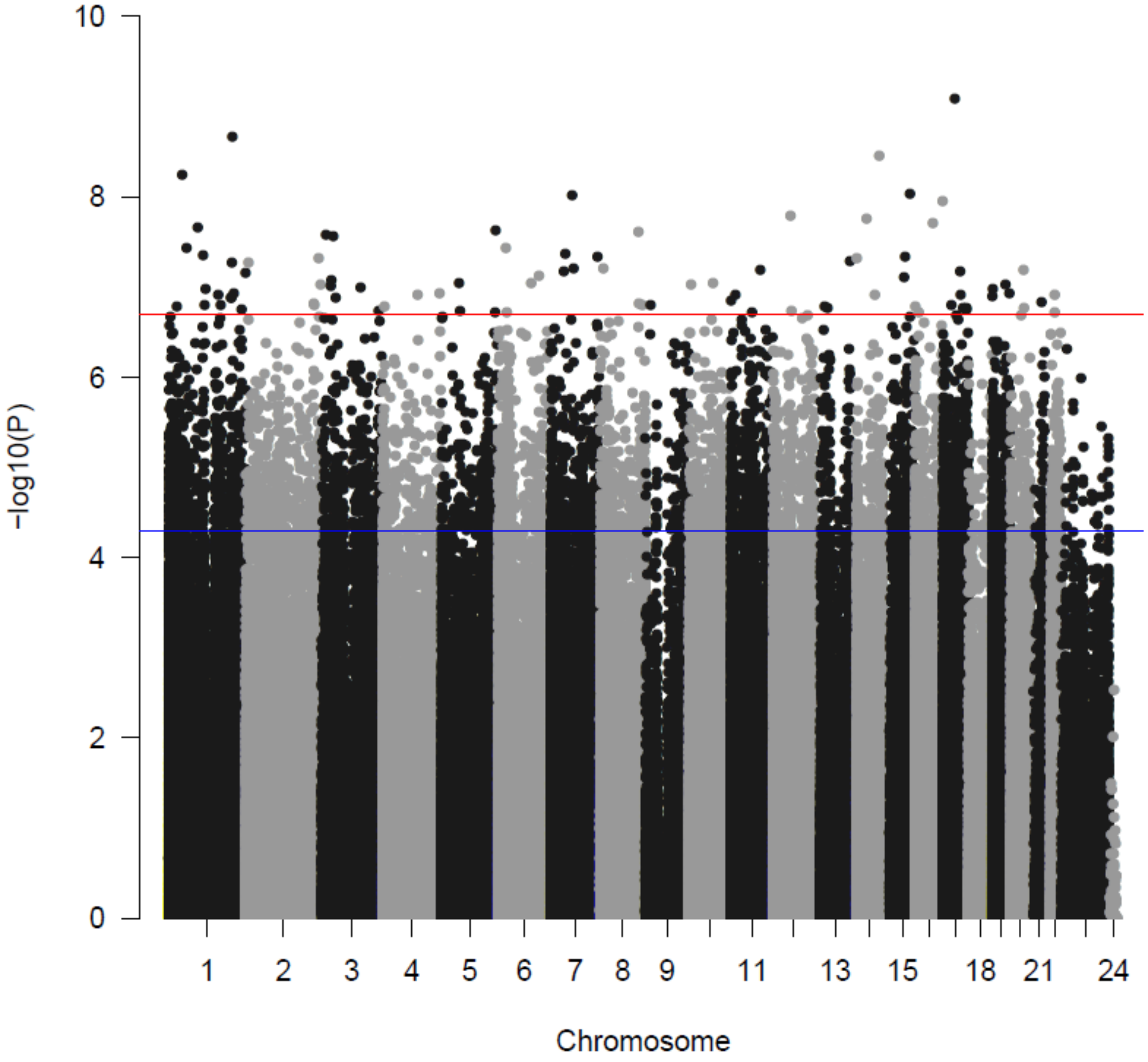

Supplementary Figure S3 –Surgery-induced change in an inflammation-related polyepigenetic score reflect perioperative levels of CRP and IL-6. A) Within each individual, the average polyepigenetic score was significantly increased at POD1 (P = 2.7E-04) and at POD4/7 (P = 3.3E-04), indicating upregulation of the immune system following surgery (Barker et al., 2018). Scatter plots show the correlation between the inflammation-related polyepigenetic score and **B)** serum CRP and **C)** IL-6.

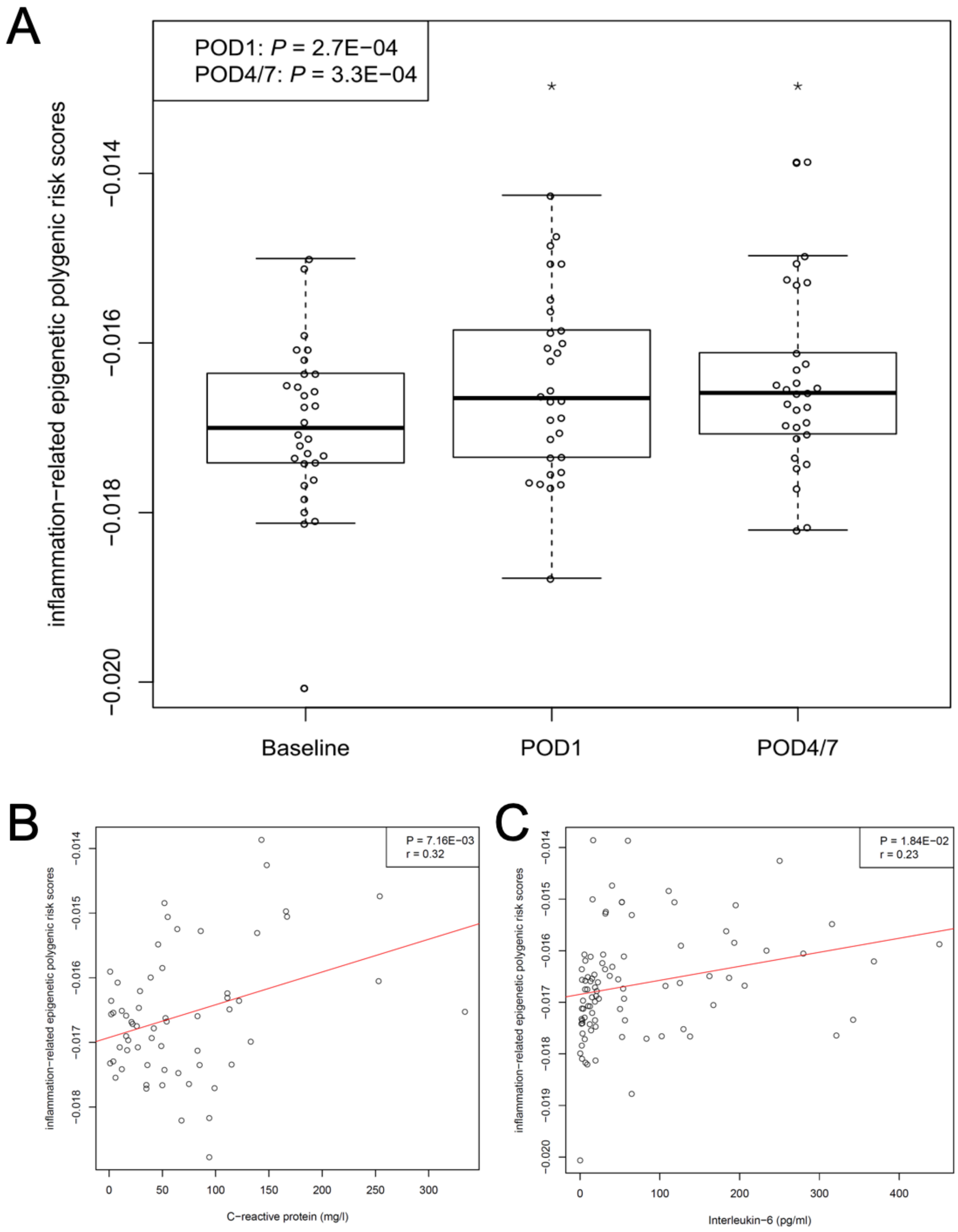

Supplementary Figure S4 – Surgery-induced change in an epigenetic biomarker of age. The average GrimAge[22] estimate was increased at POD1 compared to BL (mean change in GrimAge = 1.64 years, P = 2.57E-04). Error bar show the 95% confidence interval.

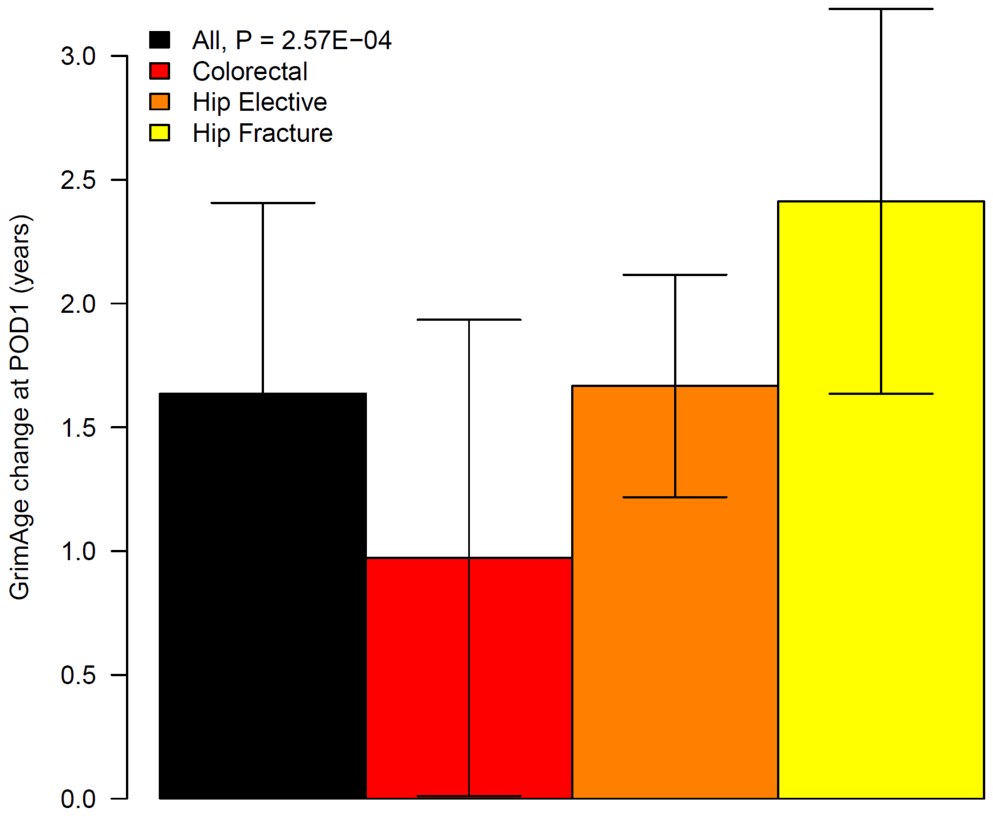

Supplementary Figure S5 – Surgery is associated with acute changes in the cellular composition of blood. Significant acute changes were observed in blood cellular composition following surgery including an increase in white blood cell count (baseline: mean white blood cell count = 9.73 *1.000/μl; POD1: mean white blood cell count = 11.35 *1.000/μl, P = 4.08E-02), neutrophil count (baseline: mean neutrophil count = 7.57 *1.000/μl; POD1: mean neutrophil count = 9.33 *1.000/μl, P = 3.00E-02), and monocyte count (baseline: mean monocyte count = 0.68 *1.000/μl; POD1: mean monocyte count = 0.85 *1.000/μl, P = 5.64E-03), and decreases in red blood cell count (baseline: mean red blood cell count = 4.04 *10E6/μl; POD1: mean red blood cell count = 3.38 *10E6/μl, P = 3.24E-09), platelets (baseline: mean platelets = 247.5 *1.000/μl; POD1: mean platelets = 212.5 *1.000/μl, P = 8.66E-07), lymphocyte count (baseline: mean lymphocyte count = 1.28 *1.000/μl; POD1: mean lymphocyte count = 1.11 *1.000/μl, P = 2.34E-02), eosinophil count (baseline: mean eosinophil count = 0.15 *1.000/μl; POD1: mean eosinophil count = 0.04 *1.000/μl, P = 4.03E-03) and basophil count (baseline: mean basophil count = 0.05 *1.000/μl.

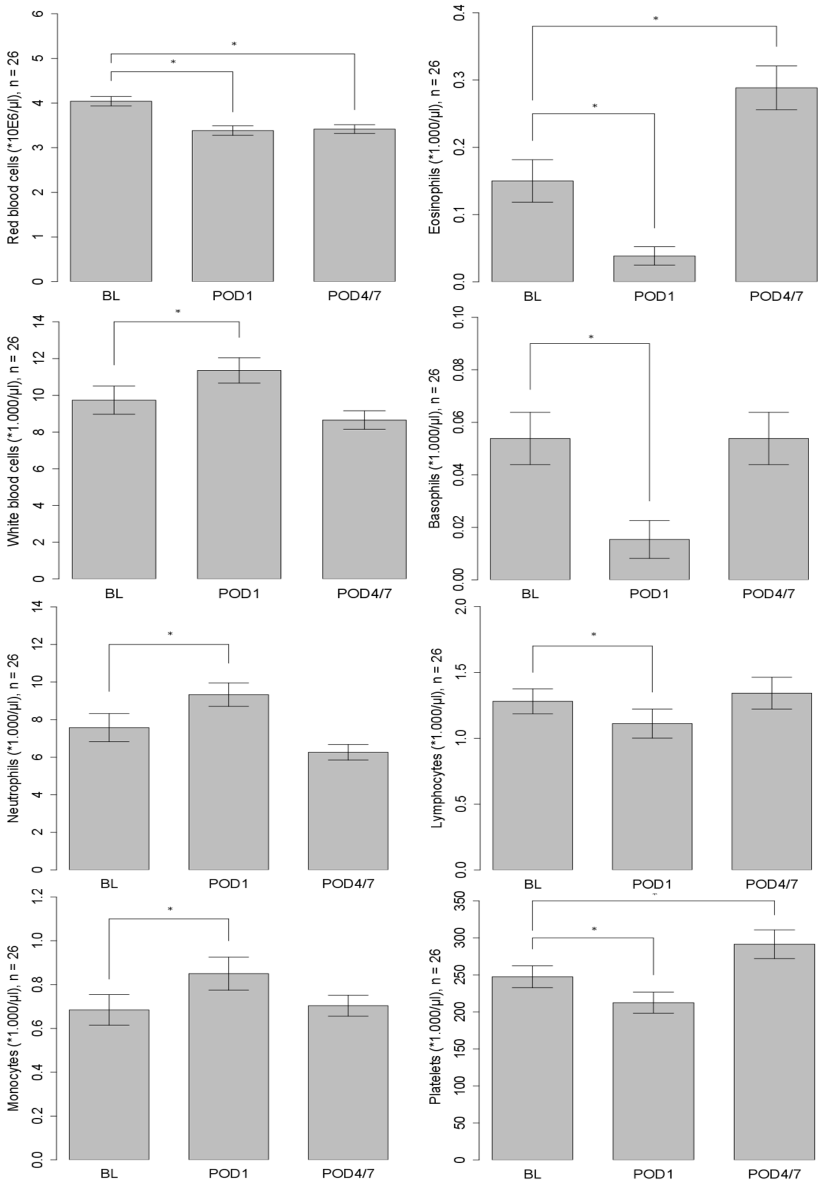

Supplementary Figure S6 – Surgery is associated with acute changes in multiple blood plasma measures. Significant acute changes were observed in plasma-based blood measures including an increase in bilirubin (baseline: mean bilirubin = 8.2 μmol/l; POD1: mean bilirubin = 13.3 μmol/l, P = 4.19E-03), and decreases in alkaline phosphatase (baseline: mean alkaline phosphatase = 82.8 iu/l; POD1: mean alkaline phosphatase = 66.9 iu/l, P = 1.15E-05), albumin (baseline: mean albumin = 44.2 g/l; POD1: mean albumin = 35.6 g/l, P = 1.19E-10), and sodium (baseline: mean sodium = 137.9 mEq/l; POD1: mean sodium = 135.7 mEq/l, P = 2.41E-04), haemoglobin (baseline: mean haemoglobin = 123.4 g/l; POD1: mean haemoglobin = 102.7 g/l, P = 5.33E-09) and haematocrit (baseline: mean haematocrit = 36.9 %; POD1: mean haematocrit = 30.6 %, P = 6.16E-09). Error bars show the 95% confidence interval. BL: baseline, POD1: postoperative day1, POD4/7: postoperative day 4 to postoperative day 7.

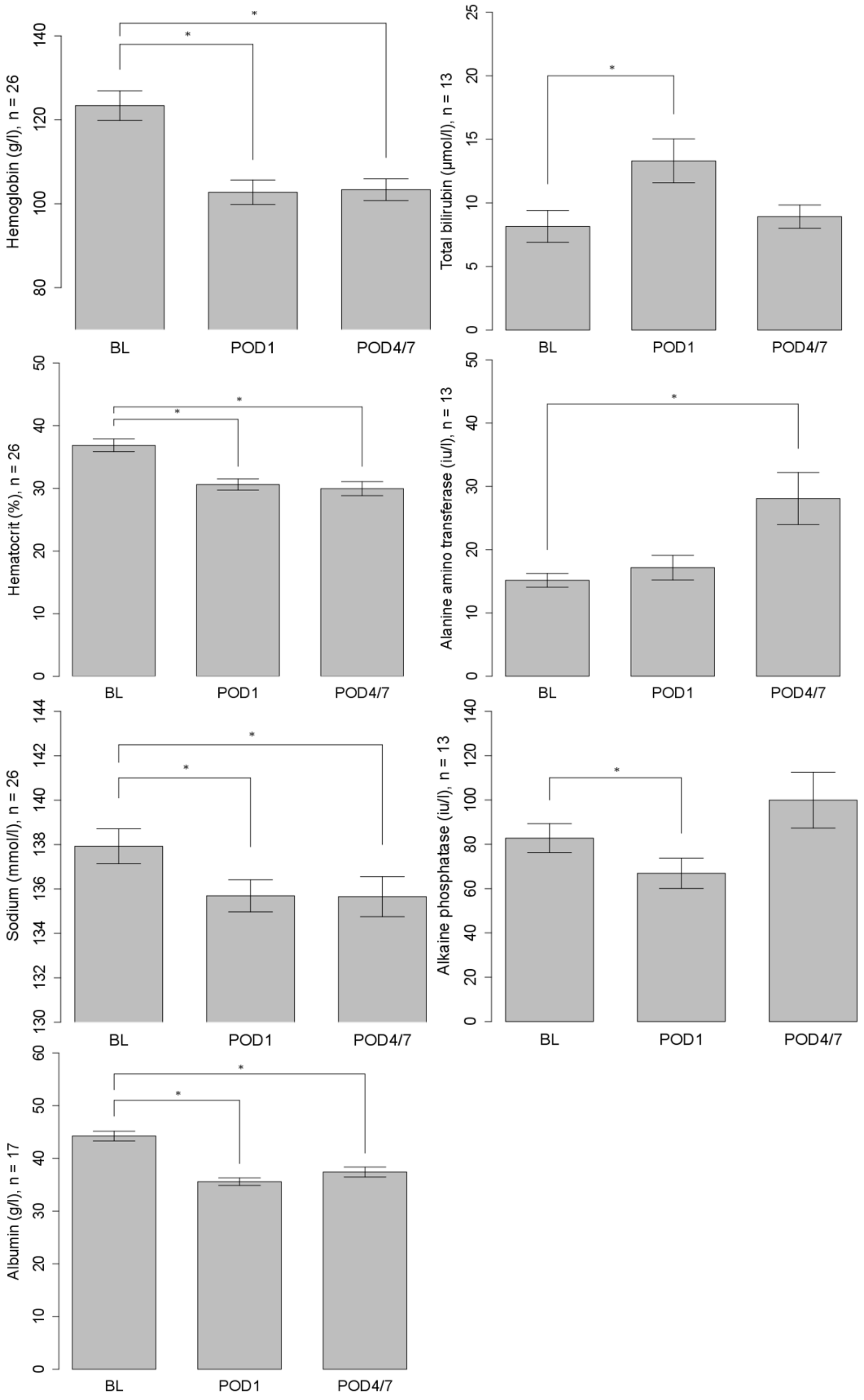

Supplementary Figure S7 – Blood cell proportion estimates derived from DNA methylation data are correlated with empirical cell-type measures. Shown is the correlation between empirically-measured ratios between monocytes and lymphocytes and those derived from DNA methylation data in peripheral mononuclear cells (n = 86, r = 0.713, *P* = 8.53E-15).

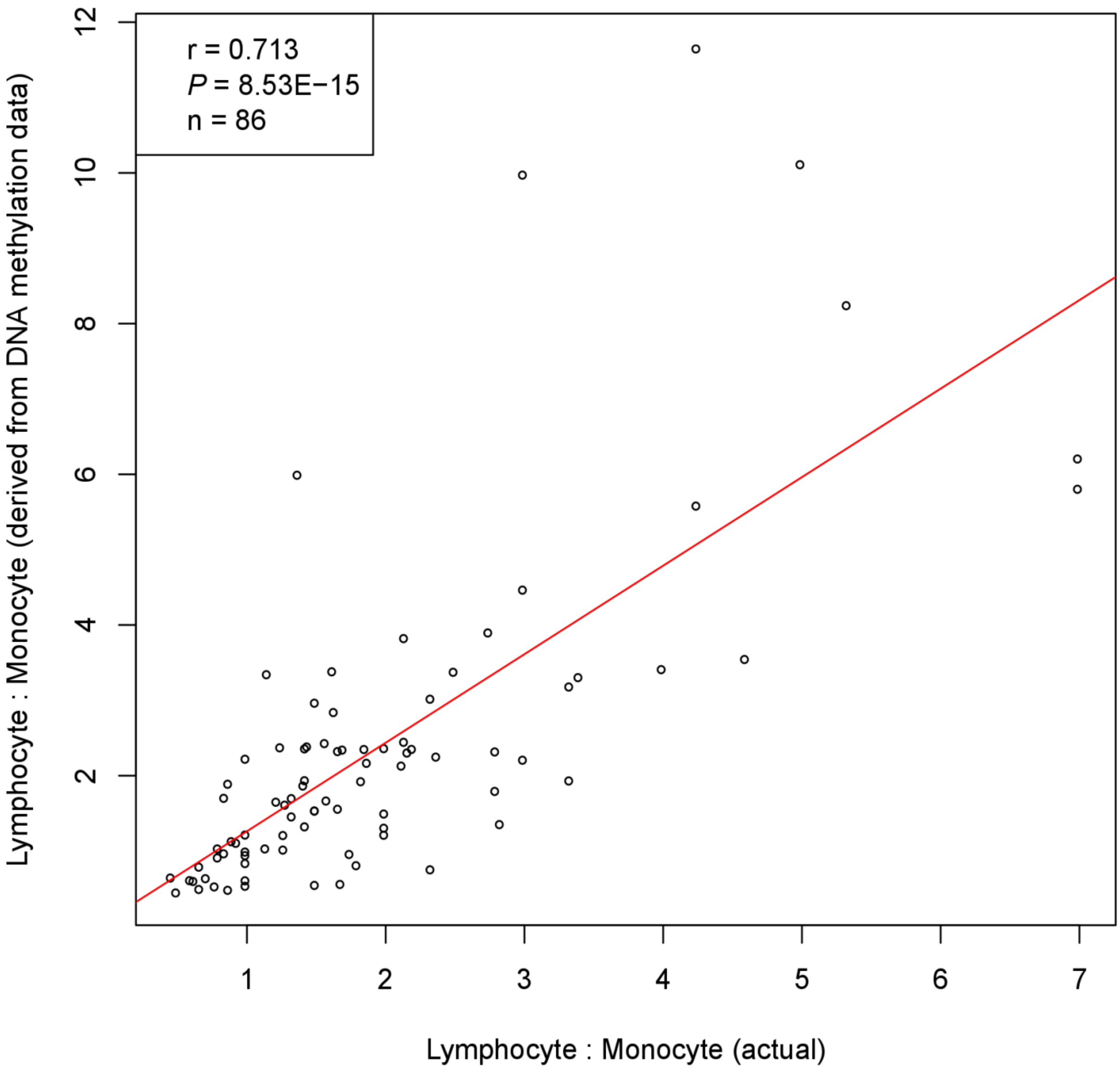

Supplementary Figure S8 - There was a strong correlation for effect sizes (r = 0.955, P = 2.20E-47) between models (corrected and uncorrected for derived cell proportions) for DMPs identified in our uncorrected model.

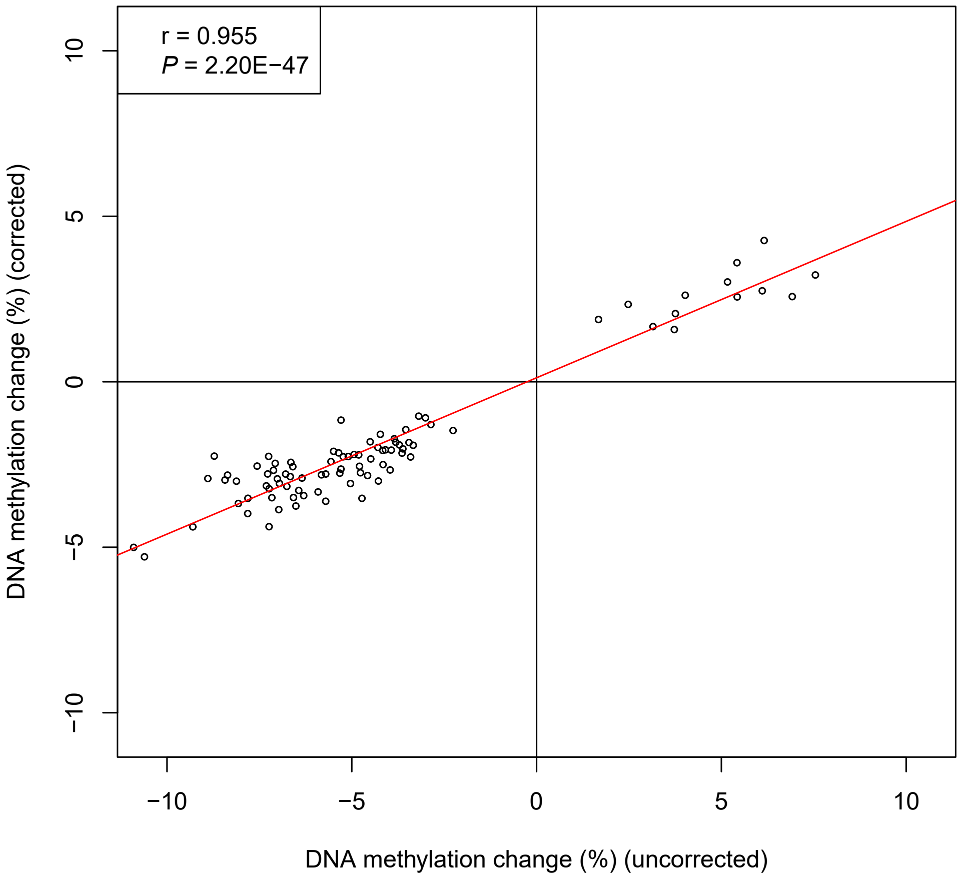

Supplementary Figure S9 – There was a strong correlation for P values (r = 0.621, P = 6.45E-11) between models (corrected and uncorrected for derived cell proportions) for DMPs identified in our model uncorrected for blood cell-type proportions.

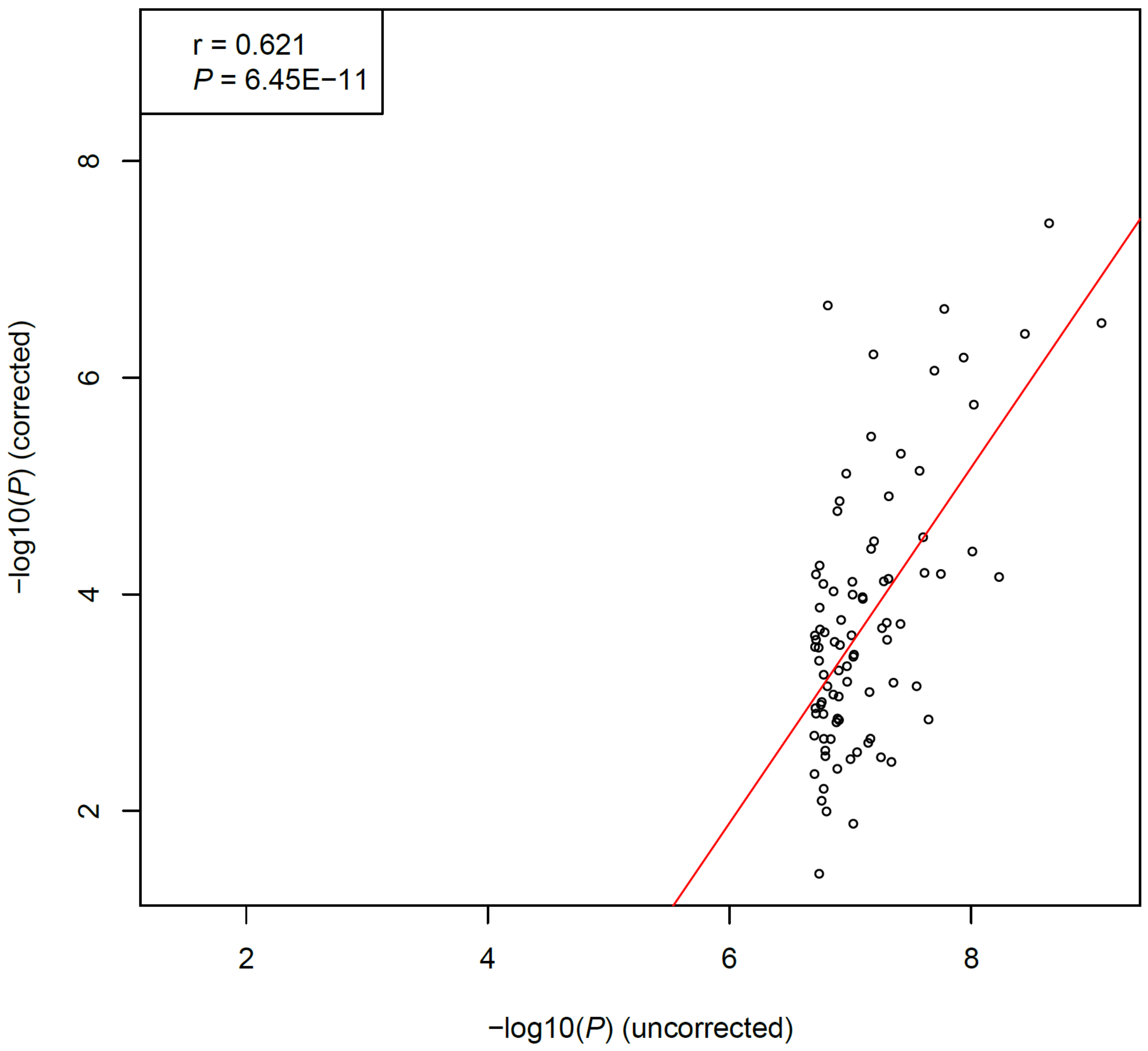

Supplementary Figure S10 – Manhattan plot for acute DNA methylation changes associated with major surgery in a model correcting for blood cell-type proportions. Within each individual, DNA methylation was compared between BL and POD1. Red line = experiment-wide significance threshold (P < 2.0E-07). Blue line = discovery significance threshold (P < 5.0E-05) used for gene ontology analysis.

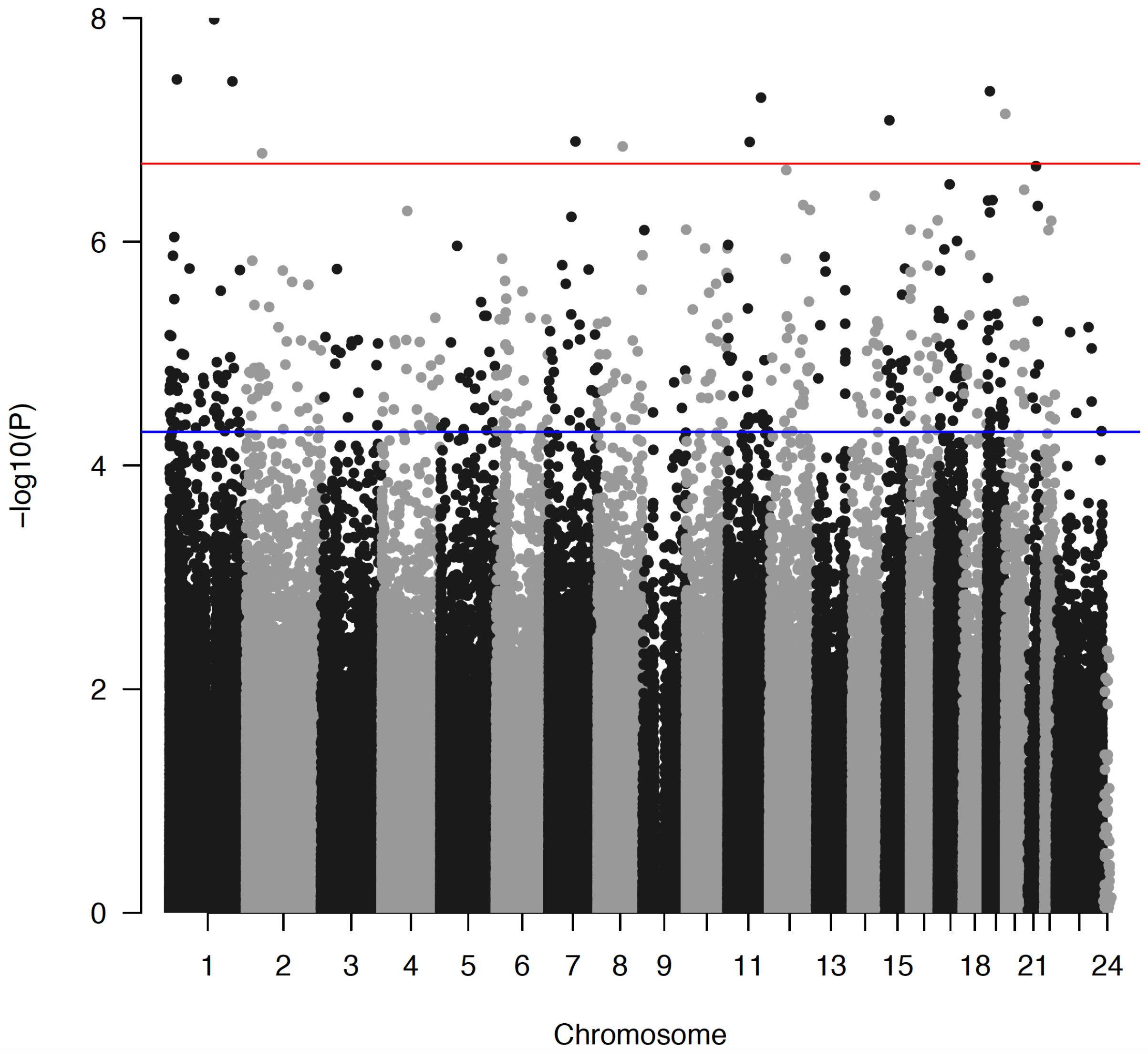

Supplementary Figure S11 - Manhattan plot for DNA methylation changes between BL and POD4/7 associated with major surgery in a model correcting for blood cell-type proportions. Red line = experiment-wide significance threshold (P < 2.0E-07). Blue line = discovery significance threshold (P < 5.0E-05) used for gene ontology analysis.

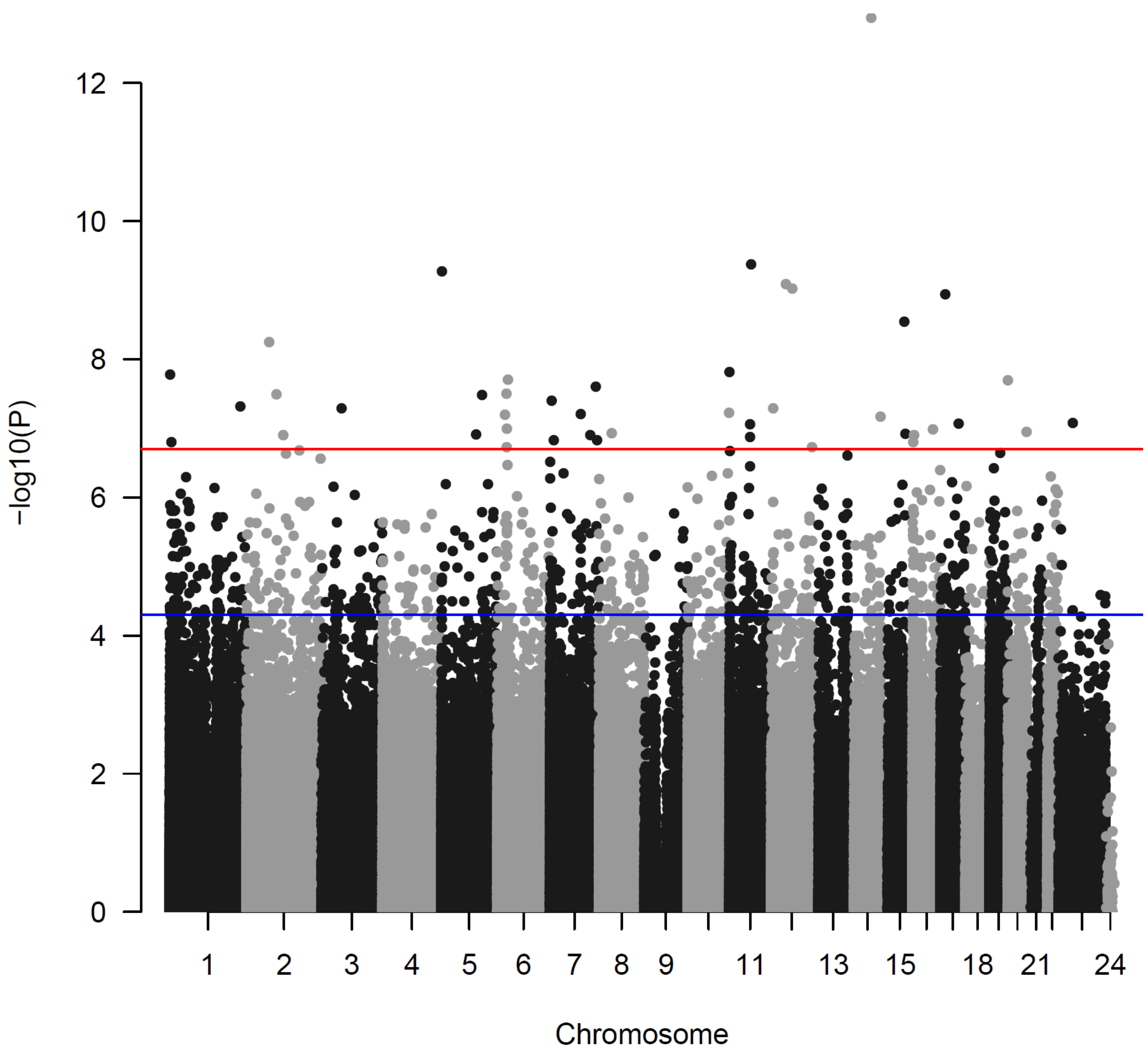

Supplementary Figure S12 – Comparison of bisulfite-pyrosequencing and Illumina 450K array data for DNA methylation at cg24501381. There was a highly significant correlation between 450K array and bisulfite-pyrosequencing datasets (P = 1.04E−23).

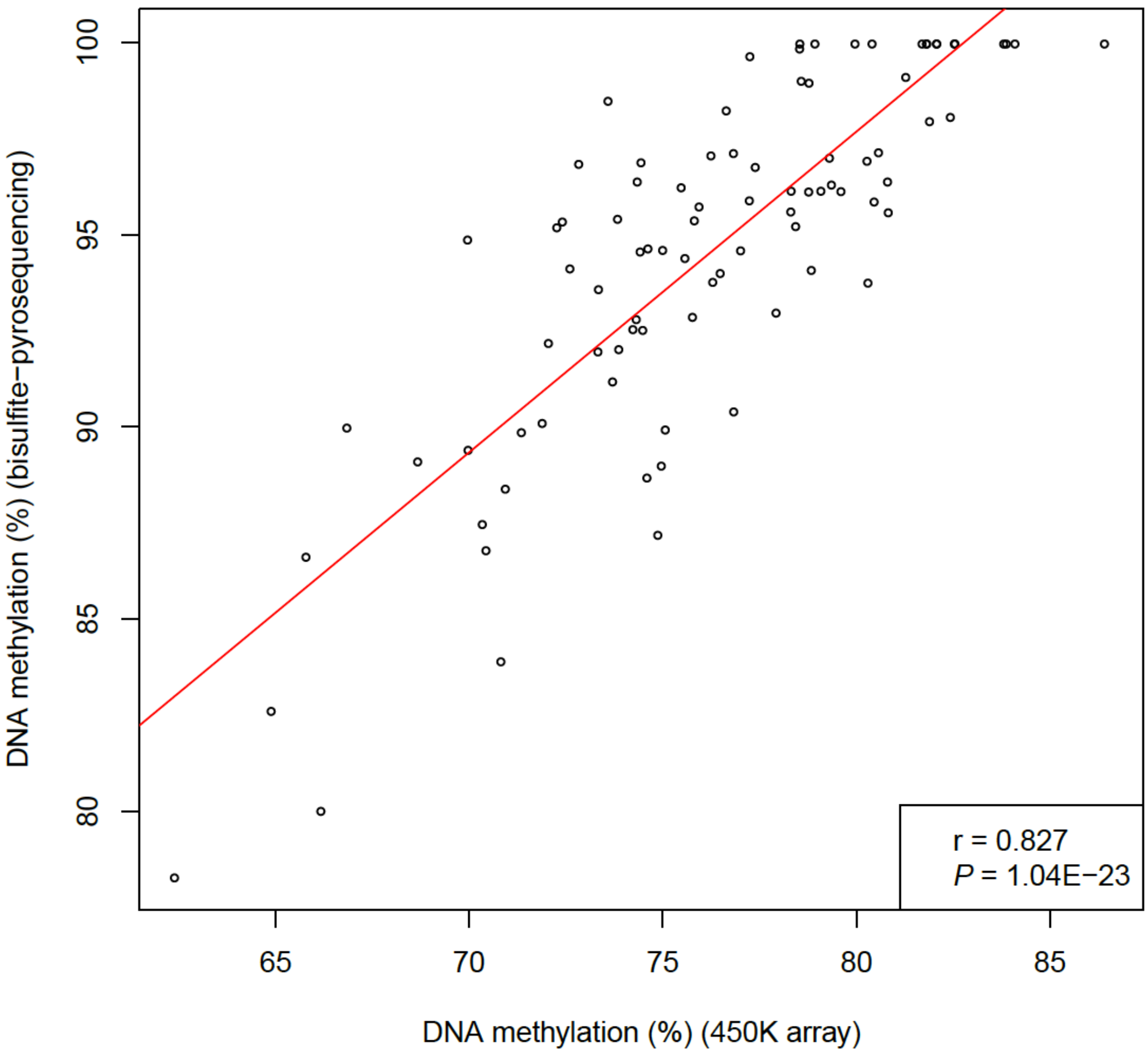

Supplementary Figure S13 – Bisulfite-pyrosequencing data confirmed a significant surgery-associated reduction in DNA methylation at cg24501381.

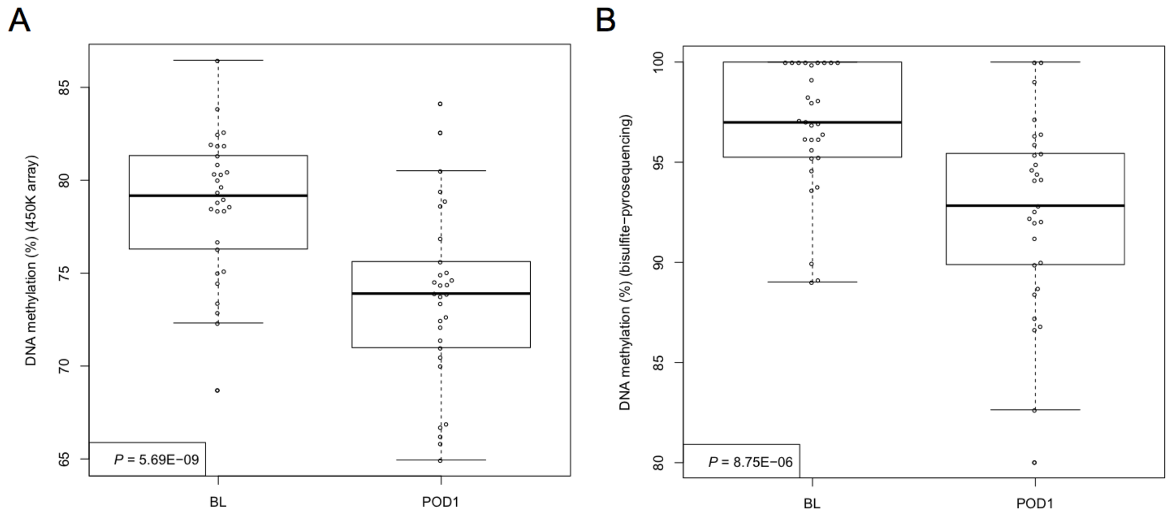

Supplementary Figure S14 – Comparison of bisulfite-pyrosequencing and Illumina 450K array data for DNA methylation at cg26344619. There was a highly significant correlation between 450K array and bisulfite-pyrosequencing datasets (P = 5.38E-19).

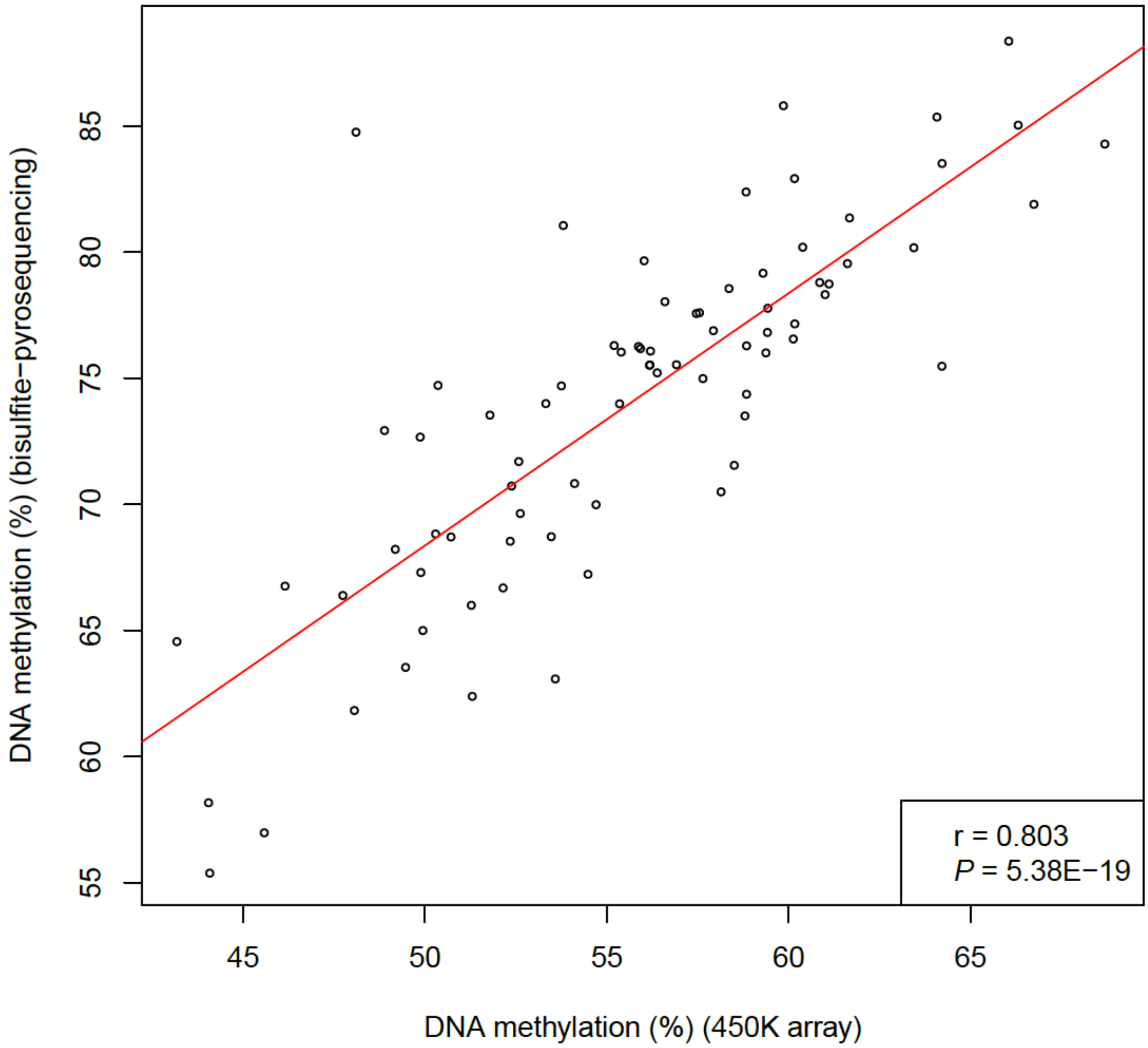

Supplementary Figure S15 – Bisulfite-pyrosequencing data confirmed a significant surgery-associated reduction in DNA methylation at cg26344619.

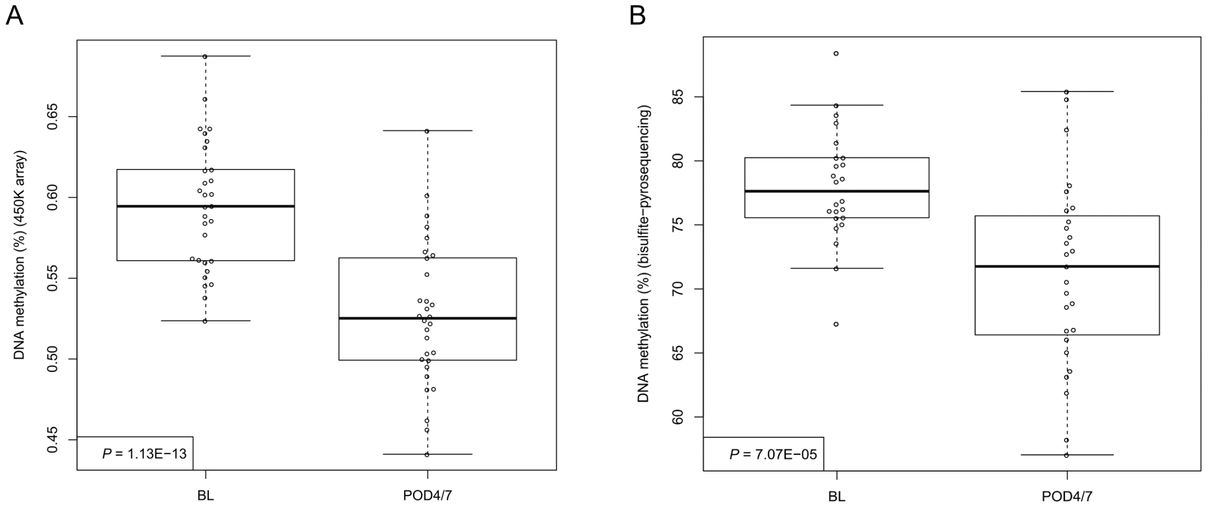

Supplementary Figure S16 - Surgery-type-specific DMPs (P < 2.0E-07) at POD1. Data for each of these eight DMPs is given in **Supplementary Table 11**.

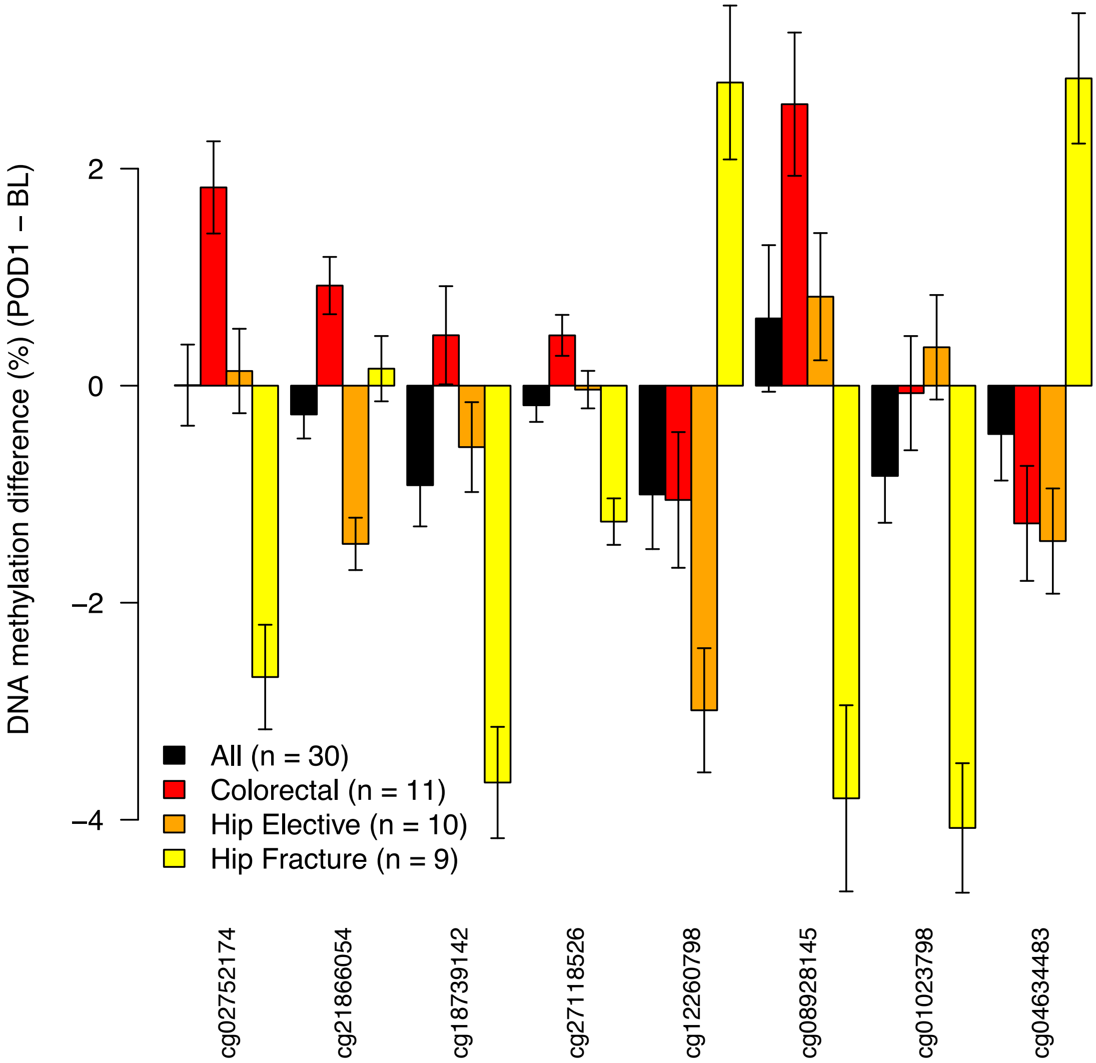

Supplementary Figure S17 - Surgery-type-specific DMPs (P < 2.0E-07) at POD4/7. Data for each of these eleven DMPs is given in **Supplementary Table 12**.

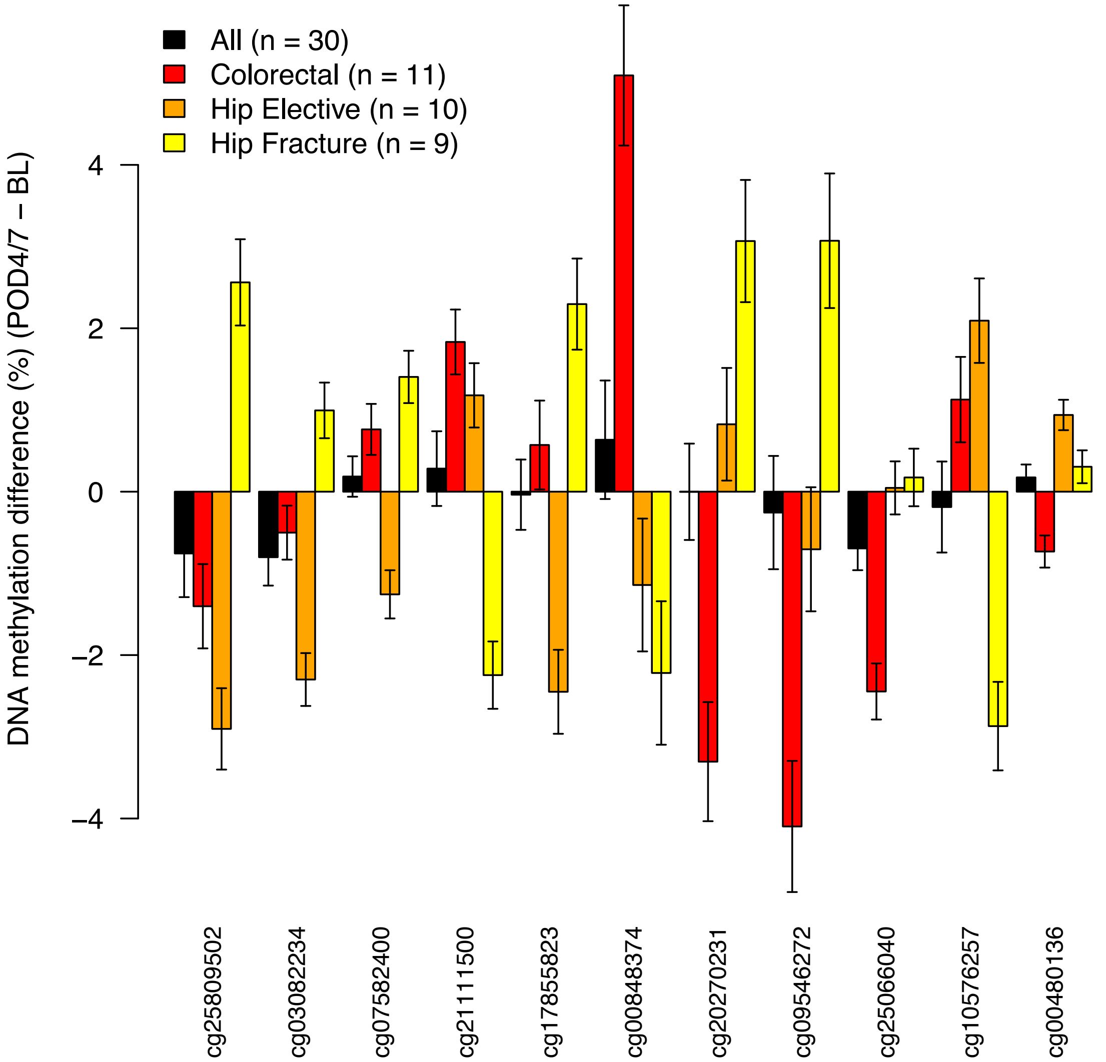

Supplement: Supplementary file 2 — Supplementary Figures [file 41598_2020_62262_MOESM2_ESM.pdf]
