# Supplementary material for: Major surgery induces acute changes in measured DNA methylation associated with immune response pathways
Source: Sci Rep. 2020 Apr 1;10:5743. doi: 10.1038/s41598-020-62262-x (PMC7113299; doi:10.1038/s41598-020-62262-x)
Supplement: Supplementary file 3 — Supplementary Table Legends [file 41598_2020_62262_MOESM3_ESM.pdf]

Supplementary Table Legends

Supplementary Table S1 - Acute changes in DNA methylation following major surgery.
Shown are DMPs ($P < 5.0E-05$) identified in an analysis comparing baseline (BL) PBMC samples to those at post-operative day 1 (POD1). DMP = differentially methylated position; POD1 = post-operative day1; Position = location of DMP (hg19); chr = chromosome; GREAT annotation = genes annotated to each DMP using GREAT with distance from transcription start site; BL DNAm = average DNA methylation level at baseline; DNAm change = average surgery-induced DNA methylation change.

Supplementary Table S2 – Differentially methylated regions characterized by acute changes following major surgery.
Sidak P-values were calculated according to Stouffer-Liptak-Kechris and Sidak correction by *comb-p*. DMR = differentially methylated region; BL = baseline; POD1 = post-operative day1; chr = chromosome; nProbes = number of probes included in specified DMR.

Supplementary Table S3 - Gene ontology (GO) term enrichment analysis on genes annotated to DMPs associated with major surgery.
GO = gene ontology; DMP = differentially methylated position; POD1 = post-operative day 1; nGenes = number of genes in each GO pathway; nTest = number of tested genes in each GO category annotated to DMPs ($P < 5.0E-5$).

Supplementary Table S4 - The acute changes in DNA methylation at sites annotated to immune system genes paralleled changes in blood cell proportions.
Shown are levels of blood serum and blood cell counts at each time-point quantified in individuals included in our DNA methylation study. Differences were identified using a paired t-test for POD1 and POD4/7 compared to BL. BL = baseline; POD1 = postoperative day 1; POD4/7 = postoperative day 4 – 7; n = number of patients.

Supplementary Table S5 – Cell proportion estimates derived from DNA methylation data highlight shifts associated with major surgery.
Alterations from baseline were tested at POD1 and POD4/7 using a paired t-test. *4 participants had missing data regarding actual levels of lymphocyte/monocyte ratios.

Supplementary Table S6 - Acute changes in DNA methylation following major surgery, correcting for derived blood cell proportions.
Shown are DMPs ($P < 5.0E-05$) identified in an analysis comparing baseline PBMC samples to those at POD1. DMP = differentially methylated position; POD1 = post-operative day1; Position = location of DMP (hg19); chr = chromosome; GREAT annotation = annotated genes with distance from transcription start site; BL DNAm = average DNA methylation level at baseline; DNAm change = average surgery-induced DNA methylation change.

Supplementary Table S7 - Differentially methylated regions characterized by acute changes following major surgery in a model correcting for derived blood cell proportions.
DMR = differentially methylated region; BL = baseline; POD1 = postoperative day1; POD4/7 = postoperative day 4 – 7; chr = chromosome; Beta = average of surgery-induced DNA methylation change. BL DNAm = average DNA methylation level at baseline; DNAm change = average surgery-induced DNA methylation change.

Supplementary Table S8 - Gene ontology (GO) term enrichment analysis on genes annotated to DMPs at POD1 following major surgery in a model correcting for blood cell heterogeneity.
GO = gene ontology; DMP = differentially methylated position; POD1 = post-operative day 1; nGenes = number of genes in each GO pathway; nTest = number of tested genes in each GO category annotated to DMPs (P < 5.0E-5).

Supplementary Table S9 – Surgery-induced changes in DNA methylation associated with shifts in serum IL-6 levels.
Shown are DMPs (P < 5.0E-05) identified in an analysis comparing baseline PBMC samples to those at POD1. DMP = differentially methylated position; POD1 = post-operative day1; Position = location of DMP (hg19); chr = chromosome; GREAT annotation = genes annotated to each DMP using GREAT with distance from transcription start site; DNAm change per 10 pg/ml = average of surgery-induced DNA methylation change per10 pg/ml IL-6.

Supplementary Table S10 - Persistent changes in DNA methylation following major surgery, correcting for blood cell heterogeneity.
Shown are DMPs (P < 5.0E-05) identified in an analysis comparing baseline PBMC samples to those at POD4/7. DMP = differentially methylated position; POD4/7 = post-operative day4 - 7; Position = location of DMP (hg19); chr = chromosome; GREAT annotation = genes annotated to each DMP using GREAT with distance from transcription start site; BL DNAm = average DNA methylation level at baseline; DNAm change = average surgery-induced DNA methylation change.

Supplementary Table S11 - Gene ontology (GO) term enrichment analysis on genes annotated to DMPs at POD4/7 following major surgery in a model correcting for blood cell heterogeneity.
GO = gene ontology; DMP = differentially methylated position; POD1 = post-operative day 1; nGenes = number of genes in each GO pathway; nTest = number of tested genes in each GO category annotated to DMPs (P < 5.0E-5).

Supplementary Table S12 – Surgery-type-specific DMPs at POD1.
Shown are DMPs (P < 2.0E-07) identified in an analysis comparing the surgery-type-specific DNA methylation change from baseline to POD1. DMP = differentially methylated position; POD1 = post-operative day1; Position = location of DMP (hg19); chr = chromosome; GREAT annotation = annotated genes with distance from transcription start site; BL DNAm = average of DNA methylation at baseline; DNAm change = average of surgery-induced DNA methylation change; Surgery type P value = P values calculated by analysis of variance among patients undergoing colorectal, hip elective, and hip fracture surgery.

Supplementary Table S13 - Surgery-type-specific DMPS at POD4/7.
Shown are DMPs (P < 2.0E-07) identified in an analysis comparing the surgery-type-specific DNA methylation change from baseline to POD4/7. DMP = differentially methylated position; POD1 = post-operative day1; Position = location of DMP (hg19); chr = chromosome; GREAT annotation = annotated genes with distance from transcription start site; BL DNAm = average of DNA methylation at baseline; DNAm change = average of surgery-induced DNA methylation change; Surgery type P value = P values calculated by analysis of variance among patients undergoing colorectal, hip elective, and hip fracture surgery.

Supplementary Table S14 - Oligo primers used in our bisulfite-pyrosequencing validation experiments.
